# Identification and Characterization of Cancer-Related Risk Metabolic Subpathways Reveal Their Functional Significance in Cancer

**DOI:** 10.3390/ijms27104246

**Published:** 2026-05-10

**Authors:** Hongying Zhao, Jinxing Yan, Ming Wu, Shiyi Li, Weiming He, Xiangzhe Yin, Wangyang Liu, Ying Liu, Meiting Fei, Wan Li, Junjie Lv, Lina Chen, Li Wang

**Affiliations:** 1 College of Bioinformatics Science and Technology, Harbin Medical University, Harbin 150081, China; zhaohongying@hrbmu.edu.cn (H.Z.); yanjinxing0712@163.com (J.Y.); wm734393188@163.com (M.W.); lsyshea@163.com (S.L.); yxzswyxgc@163.com (X.Y.); liuwangyang2022@163.com (W.L.); liuying2022020510@163.com (Y.L.); fmt2844143432@163.com (M.F.);; 2 Institute of Opto-Electronics, Harbin Institute of Technology, Harbin 150000, China; hewm@hit.edu.cn

**Keywords:** subpathways, biomarkers, metabolism

## Abstract

Cancer progression is accompanied by significant metabolic alterations. We developed a novel computational approach to identify cancer-related risk metabolic subpathways (CMSubpathway). By leveraging the topology of large-scale metabolic pathway gene networks, we initially identified metabolic subpathways and then refined them by taking into account pathway activity dysregulation, prognostic efficacy, and classification performance. We employed the CMSubpathway to extensively identify cancer-related metabolic subpathways across 21 cancer types. Ultimately, 12 risk metabolic subpathways were identified in six cancer types. Subsequently, the 12 overlapping genes of risk metabolic subpathways were identified as the core metabolic module genes. Utilizing the public CRISPR knockout screening datasets sourced from DepMap, we further supported our hypothesis that the essential roles of ADH5, ALDH1B1, and ALDH7A1 in breast cancer cell growth and development. The core metabolic module and its associated genes exhibited significant down-regulation at both the transcriptome and proteome levels based on data from tissues, blood, and single cells. The activity of this core metabolic module was associated with the immune infiltration levels of multiple immune cells, especially T cells. Notably, an abnormal core metabolic module was observed in CD8 T cell subtypes, with the stem-like CD8 T cell subtype showing high metabolic activity and exhaustion markers. Thus, we established a method for identifying risk metabolic subpathways in cancers, which helps to identify more precise biomarkers for cancer patients.

## 1. Introduction

The reprogramming of metabolism, the process of tumor cells reprogramming metabolic pathways to meet the energy and biosynthetic demands of tumor cells in order to adapt to hypoxia and nutrient deficiency, is a hallmark of malignancy with great potential for prognosis and targeted therapy [1]. For example, the abnormal activation and reprogramming of multiple key metabolic pathways, such as glycolysis, fatty acid synthesis, and amino acid metabolism, are considered to be one of the key factors promoting tumor progression in breast cancer [2]. Recently, an increasing number of studies have reported that abnormalities in mRNA, proteins, and metabolites play important roles in metabolic reprogramming in cancer. For instance, the study by Torresano et al. identified the up-regulation of the glucose transporter GLUT1 in cancer as a mechanism to increase glucose uptake to support tumor growth. Consistently, the overexpression of GLUT1 is associated with increased tumor aggressiveness and poor survival in patients with colon, hepatocellular, esophageal squamous cell carcinoma, and non-small lung cancer [3]. The up-regulation of the IGF2BP3 mRNA and protein expression regulates nicotinamide metabolism, which could alter oxidative phosphorylation and promote acquired resistance to EGFR inhibitors [4]. In addition, cancer metabolic reprogramming not only promotes tumor growth and metastasis but is also closely related to the immune response in the tumor microenvironment, which affects the progression of tumors and the prognosis of patients. Studies have shown that abnormal activation of glucose metabolism promotes tumor cell survival and proliferation through the Warburg effect and activates M2-like macrophages through lactate production, leading to tumor immunosuppression [5,6,7]. Therefore, understanding how reprogrammed metabolism supports the growth and evolution of specific tumor cells and identifying which reprogrammed metabolic activities are most relevant to therapeutic vulnerabilities can help reveal the mechanism of cancer progression.

Traditional pathway identification methods, using databases such as KEGG, Reactome, Pathway Commons, and WikiPathways, rely on abstract concepts built on whole biological concepts, such as apoptosis or the cell cycle. Moreover, at the genomic and transcriptomic levels, no unique mechanistic results are provided for the activity of the whole pathway, and the experimental results are indicative and of limited utility. In the context of cancer, it is not a universal dysfunction of all genes within a pathway; rather, it is typically a subset of genes that exhibits aberrant functions within specific segments of the pathway. These specific parts, known as subpathways, which are local gene subregions within biological pathways, have drawn significant attention. Metabolic subpathway identification focuses on specific branches or submodules in a pathway, which can more accurately locate metabolic processes related to specific biological functions, and decompose the complex metabolic network into simpler and clearer subunits [8]. Abnormalities in metabolic subpathways have been reported to be associated with the occurrence, progression, and prognostic outcomes of cancer [9]. For example, (S)-2,3-Epoxysqualene, located in the sterol biosynthesis subpathway, which holds a central and crucial position, is likely to have a more significant impact on the entire pathway and thus potentially has a high association with glioblastoma [10]. The identification of cancer-related subpathways not only benefits the understanding of carcinogenesis and cancer progression, but also helps to identify more precise biomarkers for cancer patients. Cancers were highly heterogeneous diseases that had great differences in transcriptome, survival prognosis and other aspects. How to effectively integrate metabolic pathways and multiple omics data to accurately identify abnormal metabolic subpathways in cancer is a challenge. In this paper, we proposed a computational approach to identify cancer-related risk metabolic subpathways based on the topology of large-scale metabolic networks and cancer-specific multi-omics. Moreover, we applied this algorithm to identify risk metabolic subpathways in multiple cancer types. We comprehensively characterized cancer-related metabolic subpathways and exhibited expression perturbations in cancer, which were significantly correlated with immune cell infiltration and prognosis. Thereby, the metabolic subpathways identified by our method are crucial for cancer, providing novel insights for immunotherapy, drug response, and prognosis assessment.

## 2. Results

### 2.1. Identification of Cancer-Related Risk Metabolic Subpathways Across Cancer Types

**Figure 1:**
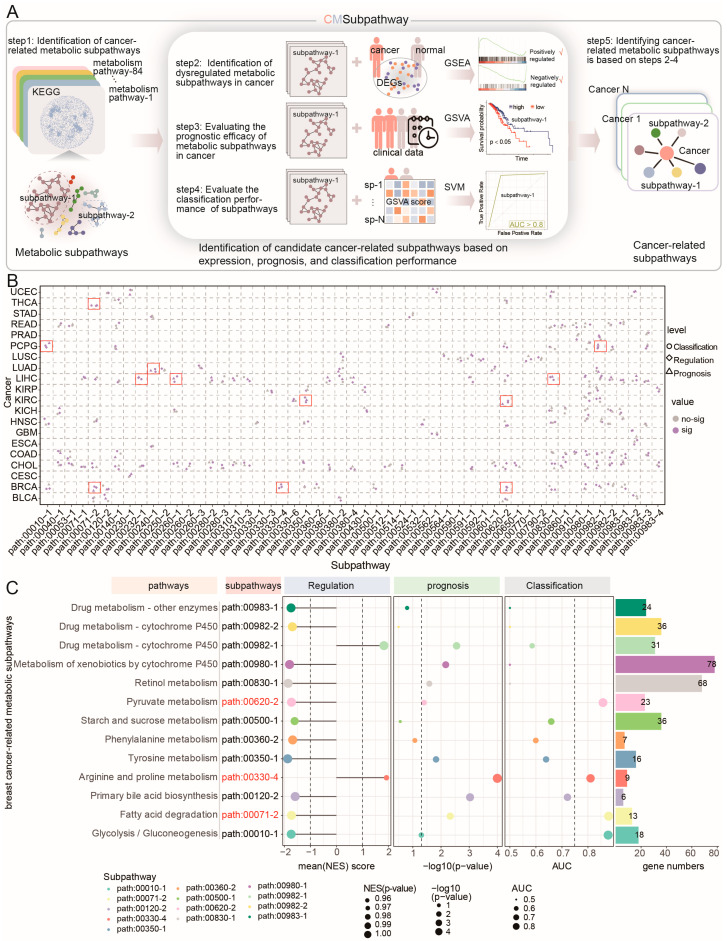
Schematic overview of the method for identifying cancer-related metabolic subpathways. (**A**) Schematic workflow. (**B**) Distributions of all cancer-related metabolic subpathways in pan-cancer. The red boxes highlight the 12 cancer-related metabolic subpathways identified across six specific cancer types (BRCA, KIRC, LIHC, LUAD, PCPG, and THCA). (**C**) Bubble map of a BRCA-related metabolic subpathway (red) at the regulation (**left**), prognosis (**middle**), disease classification (**right**) levels, and bar chart of the number of genes within the subpathway. The bubble size indicates the size of the value. The colors represent different subpathways. The mean (NES) score indicates the activity of the subpathway in BRCA patients, −log10(*p*-value) indicates whether the prognosis of the subpathway is significant, and AUC indicates the prediction result of the classification efficiency of the subpathway.

To systematically identify subpathway regions of abnormal metabolic pathways that may contribute to complex human diseases, we developed a novel computational approach, CMSubpathway, to identify cancer-related risk metabolic subpathways (Figure 1A). First, 84 metabolism-related pathways were downloaded from the KEGG database and then transformed into interaction networks. The metabolic subpathways were identified based on the LTE algorithm, which is a fast local expansion algorithm for discovering communities in large-scale networks. Second, genes that were differentially expressed between cancer samples and normal samples (|logFC| ≥ 1 and *p* < 0.05) were identified, and standardized enrichment scores (NESs) for each metabolic subpathway were calculated based on gene set enrichment analysis (GSEA). The subpathways with an absolute NES > 1, *p* < 0.05 and FDR < 0.25 were considered dysregulated metabolic subpathways in cancer. Third, candidate subpathways were identified as prognostic markers for each cancer. The GSVA score for each subpathway in every sample was calculated, and the samples were categorized into high-risk and low-risk groups based on their GSVA scores. The log-rank test was applied to evaluate the differences between the two groups, with *p* < 0.05 considered significant. Fourth, the classification performance of candidate subpathways was evaluated. An SVM model was constructed to assess subpathway classification efficiency by using the GSVA score. An AUC > 0.75 was considered efficient. Finally, we defined cancer-related metabolic subpathways that are dysregulated in cancer, correlate with prognosis, and facilitate the classification of normal and cancer samples.

Taking advantage of the expression and clinical data in The Cancer Genome Atlas (TCGA) and metabolic pathways in KEGG, the CMSubpathway was carried out to extensively identify cancer-related risk metabolic subpathways across 21 different cancer types. We identified 12 cancer-related metabolic subpathways across six types of cancer: breast cancer (BRCA), kidney renal clear cell carcinoma (KIRC), liver hepatocellular carcinoma (LIHC), lung adenocarcinoma (LUAD), pheochromocytomas and paragangliomas (PCPG), and thyroid carcinoma (THCA) (Figure 1B). Specifically, we identified three BRCA-related risk metabolic subpathways, including hsa00620_2 (pyruvate metabolism pathway), hsa00330_4 (Arginine and proline metabolism pathway), and hsa00071_2 (fatty acid degradation; Figure 1C, Table S1). The subpathway hsa00620_2 was shared by BRCA and KIRC, while hsa00071_2 was shared by BRCA and THCA. For example, the low activities of the risk metabolic subpathways hsa00620_2, hsa00071_2, and hsa00330_4 were associated with a poor prognosis in BRCA (for hsa00620_2, *p* = 4.9 × 10^−2^; for hsa00071_2, *p* = 4.7 × 10^−3^; for hsa00330_4, *p* < 1.0 × 10^−4^; Figure S1A). It is consistent with our findings that the inhibition of pyruvate metabolism was sufficient to impair collagen hydroxylation and consequently the growth of breast cancer-derived lung metastases in different mouse models [11]. Decreased activity of fatty acid metabolism genes was observed in RCC tumors and was described as a feature of ccRCC [12]. In conclusion, CMSubpathway is capable of effectively identifying cancer-related risk metabolic subpathways and uncovering the commonalities and specificities of cancer metabolic reprogramming, thereby offering novel insights for further clinical research.

### 2.2. Independent Dataset Validation

**Figure 2:**
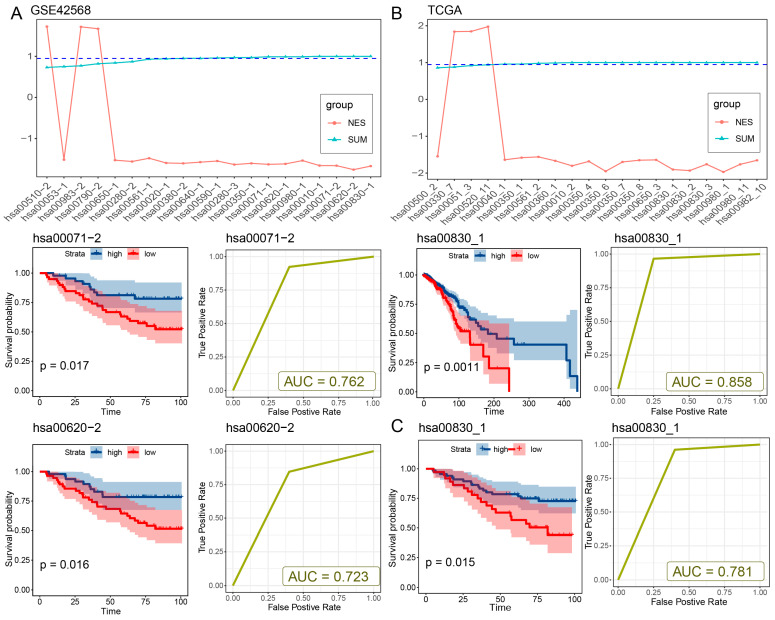
Independent dataset validation. (**A**) The NES average of 13 subpathways was calculated in 100 repetitions in the GSE42568 dataset (**top panel**). The blue dashed lines indicate the significance threshold for the NES. Kaplan–Meier survival plots and ROC curves of the two subpathways with fine classification performance among these subpathways in the GSE42568 dataset (**down panel**). The dysregulation of expression, prognostic discrimination, and differential identification between normal and diseased samples of subpathways identified by the Subpathway-CorSP algorithm in the (**B**) TCGA-BRCA dataset and (**C**) GSE42586 data set.

Since breast cancer has the largest number of risk metabolic subpathways and shares a relatively large number of subpathways with other cancers, we take breast cancer as an example to verify the effectiveness of the method. An independent validation dataset, which contains matched RNA-seq and survival data, was obtained from the GSE42568 dataset, consisting of 104 breast tumor samples and 17 normal samples. The same cancer-related risk metabolic subpathway identification method was applied to the validation dataset. In line with the outcomes from the TCGA-BRCA dataset, the down-regulation of two out of the three risk metabolic subpathways (hsa00071-2 and hsa00620-2) within tumor samples exhibited a significant correlation with survival prognosis (log-rank *p* = 0.017 and 0.016, Figure 2A, Table S2). Elevated expression levels of these subpathways were associated with a decreased risk of adverse outcomes. Furthermore, these subpathways exhibited fine classification performance in distinguishing between normal and diseased samples (AUC = 0.76 and 0.72, Figure 2A, Table S2). We regarded the subpathways hsa00071-2 and hsa00620-2 that were consistent between the two datasets as the breast cancer risk metabolic subpathways. Moreover, to enhance the confidence of our method, we utilized the Subpathway-CorSP algorithm [13] instead of the LTE algorithm in our approach to validate the identified metabolic subpathways within the TCGA-BRCA and GSE42568 datasets. The TCGA-BRCA dataset and GSE42568 dataset consistently identified one risk metabolic subpathway (hsa00830_1) that was significantly down-regulated and associated with a high risk due to low expression (*p* < 0.05, Tables S2 and S3). The subpathway hsa00830_1 was significantly down-regulated in patients and showed significant prognostic value (log-rank *p* = 1.5 × 10^−2^) in both the TCGA-BRCA and GSE42568 datasets, effectively distinguishing between normal and cancer samples (AUC = 0.858 in TCGA and AUC = 0.781 in GSE42568; Figure 2B,C and Figure S1B, Tables S3 and S4). The subpathway hsa00830_1 has a significant intersection of metabolic genes with breast cancer risk subpathways hsa00071-2 and hsa00620-2 (hypergeometric test, *p* = 3.17 × 10^−10^ for hsa00071-2 and *p* = 4.04 × 10^−8^ for hsa00620-2) by our method using the LTE algorithm, including shared genes ADH1A, ADH1B, ADH1C, ADH4, ADH5, ADH6, and ADH7 (Figure S1C, Tables S2 and S3). Furthermore, to assess the performance of CMSubpathway, we performed a comparison with several established subpathway analysis methods, including Subpathway-CorSP, LTE, and SubpathwayGMir. Predictive accuracy and robustness were assessed across the aforementioned 11 independent validation datasets. The results demonstrated that our methodology achieved substantially higher predictive accuracy (mean AUC = 0.95) and greater algorithmic robustness than LTE (mean AUC = 0.917), Subpathway-CorSP (mean AUC = 0.924) and SubpathwayGMir (mean AUC = 0.898) across all tested datasets (Figure S1D). These results further validated the feasibility and reliability of our proposed method.

### 2.3. Metabolic Crosstalk of the Risk Metabolic Subpathway Driven by the Core Module in BRCA

#### 2.3.1. Consistent Dysregulation of the Core Metabolic Module at Multiple Levels

**Figure 3:**
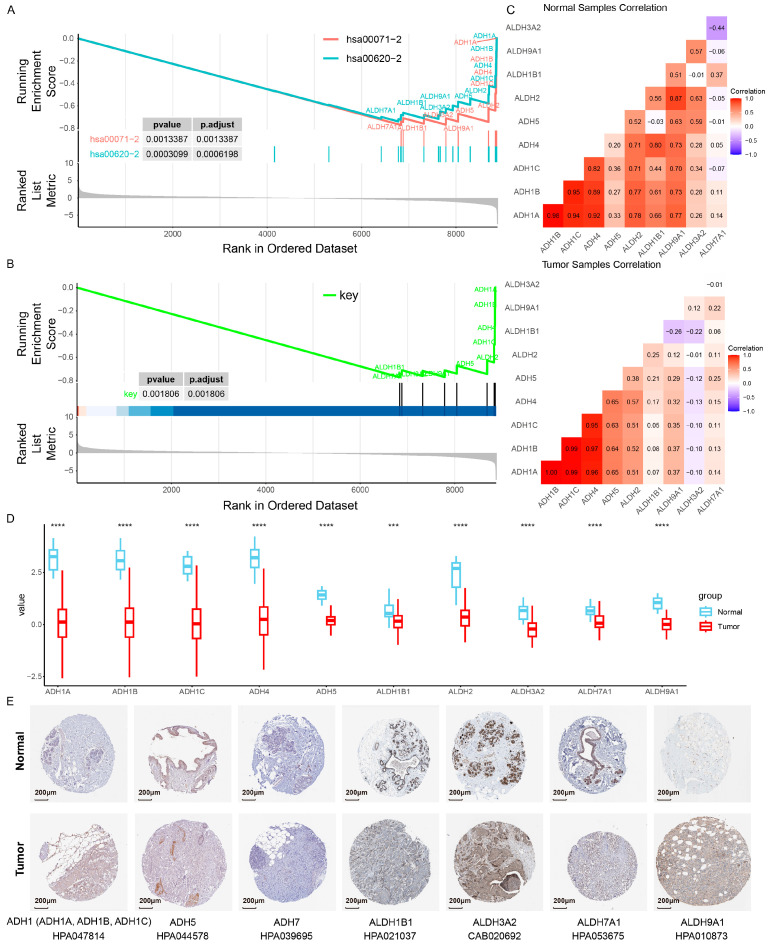
Protein level validation of subpathways. Pathway activity of hsa00071-2 and hsa00620-2 (**A**), as well as that of the core metabolic module (**B**), was analyzed using protein data. (**C**) Heatmap of the correlation of protein expression between the key genes in normal samples (**upper panel**) and tumor samples (**lower panel**). (**D**) Box plots show the differences in protein levels of the key genes between normal and tumor samples. ***: *p*< 0.001, ****: *p* < 0.0001. (**E**) The protein expression of 9 key genes was down-regulated in breast cancer tissues compared with that in normal tissues based on the HPA database. The lower part of the panel displays the gene and antibody numbers.

Based on the significant intersection of metabolic genes in the breast cancer risk metabolic subpathways hsa00071-2 and hsa00620-2 identified above (*p* = 3.12 × 10^−23^), we identified a set of 12 overlapping genes. Biologically, these genes (predominantly encoding ADH and ALDH family enzymes) reside at the critical topological crossroads linking fatty acid degradation and pyruvate metabolism, facilitating essential metabolic crosstalk. Given their key roles in bridging these distinct metabolic processes, we defined this 12-gene set as a breast cancer-related core metabolic module (Figure S1E). We found that the core metabolic module genes exhibit consistent down-regulation at the transcriptome and proteome levels in breast cancer patients. Specifically, at the transcriptomic level, through analyzing the results of differentially expressed genes from TCGA, GSE42568 and the gene expression data of breast cancer from the cancer atlas of metabolic profiles (CAMP), the core metabolic module genes consistently showed significant down-regulation results in tumor patients (Figure S2A,B). Additionally, we collected two independent breast cancer clinical datasets from the Expression Atlas database. The first dataset (E-GEOD-45581) comprises 40 untreated breast cancer patient samples and 5 normal controls. The second dataset (E-MTAB-779) includes 20 untreated breast carcinoma patient samples and 22 normal controls. These two breast cancer clinical datasets were used to validate the expression differences in core metabolic module genes. The results showed that most core metabolic module genes were down-regulated in breast cancer tissues relative to normal tissues (Figure S2C). Furthermore, we also investigated the expression of the genes associated with this core module within the peripheral blood cohort of breast cancer patients and discovered that they were down-regulated across multiple sets of blood samples. For example, ALDH3A2, ALDH2, ALDH9A1, ADH5, and ALDH7A1 had significantly lower expression in multiple cancer blood sample cohorts (Figure S2D, Table S4). At the proteomic level, we obtained the protein expression data from the CPTAC dataset, which includes 18 normal and 218 BRCA samples, and conducted a differential analysis. Consistent with the RNA-seq results, these two risk metabolic subpathways and their core metabolic module were significantly down-regulated at the protein level in breast cancer samples (Figure 3A,B), which revealed a congruent down-regulation, corroborating the RNA-seq level findings. These 12 key genes also exhibited a correlation pattern at the protein level that mirrored the RNA-seq data, with ADH1A, ADH1B, and ADH1C demonstrating pronounced correlations in both tumor and normal samples (Figure 3C). Additionally, the protein expression levels of the core metabolic module genes were significantly down-regulated in tumor tissue samples, which is consistent with the findings at the RNA-seq level (Figure 3D). To further validate our results, we searched the HPA database for the IHC data of the proteins encoded by these 12 key genes bridging the risk subpathways in both normal and tumor tissue sections. Through the analysis using the HPA database, it was found that, among the 12 key genes, the protein expression levels of 9 were decreased in breast cancer tissues relative to normal tissues (Figure 3E). In conclusion, we observed the dysregulation of genes within this core metabolic module at multiple levels, which might contribute to the metabolic reprogramming in breast cancer.

#### 2.3.2. Dysregulation of the Core Module Is Associated with Metabolic Reprogramming

**Figure 4:**
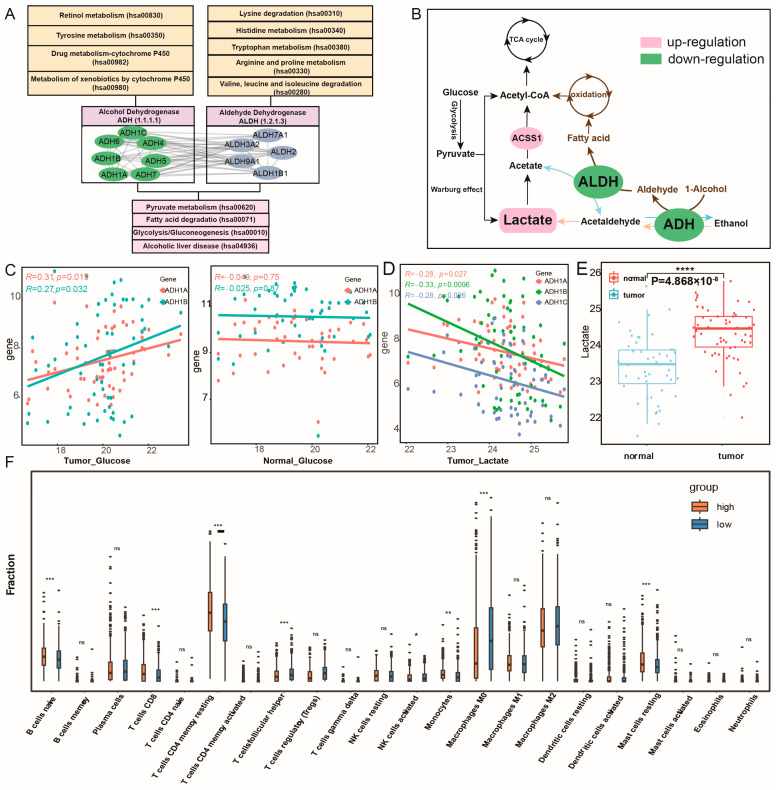
The core metabolic module plays essential roles in metabolic regulation in breast cancer. (**A**) The protein–protein interaction (PPI) network of the core metabolic module genes (categorized into ADH and ALDH families) and their roles as critical hubs linking multiple upstream and downstream metabolic pathways. (**B**) Key metabolic subpathway gene modules regulate the specific processes of glucose and fatty acid energy metabolism pathways. (**C**) The Pearson correlation coefficients between the gene expression of ADH1A and ADH1B and the glucose levels in tumor and normal samples. (**D**) The Pearson correlation coefficients between the gene expression of ADH1A, ADH1B, and ADH1C and the lactate levels. (**E**) There is a significant difference in lactate levels between tumor samples and normal samples. (**F**) The difference in immune infiltration between the high and low metabolism activity groups. ns, *p* > 0.05; *, *p* < 0.05; **, *p* < 0.01; ***, *p* < 0.001; ****, *p* < 0.0001.

These core metabolic module genes jointly regulate pyruvate metabolism, glycolysis/gluconeogenesis, and fatty acid degradation, affecting metabolic crosstalk and reprogramming (Figure 4A and Figures S3–S5). The genes within this module can be divided into ALDH and ADH families according to their regulatory roles in metabolism, which are derived from the aldehyde dehydrogenase and alcohol dehydrogenase gene families and exhibit strong internal correlation (Figure 4B and Figure S6A). To further investigate whether these key genes play a central role in subpathway crosstalk, we carried out a functional enrichment analysis of the differentially expressed gene sets in breast cancer, with and without their being considered separately. The results of our analysis demonstrated that only the gene sets that included this core metabolic module were significantly enriched in the pyruvate metabolism pathway (*p* = 2.63 × 10^−4^) and the fatty acid degradation pathway (*p* = 1.33 × 10^−4^), whereas the gene sets without these key genes did not show such significant enrichment (Figure S6B). By utilizing the gene expression and metabolites data of breast cancer, which encompassed 61 cancer samples and 47 normal samples from the CAMP [14], we found that the expression levels of ADH1A, ADH1B, and ADH1C were significantly down-regulated and positively correlated with glucose levels in cancer samples (Pearson correlation test, with *p* < 0.05 and Pearson correlation coefficient > 0). However, such a correlation was not detected in normal samples (Figure 4C and Figure S2B). This finding implies that the ADH genes within this module might be related to the high glucose consumption trait in breast cancer. Glucose serves as the principal source of lactate in the body. Glycolysis triggers the production of lactate, and lactate acts as the main fuel for the tricarboxylic acid cycle (TCA cycle), supplying energy for tumor cell respiration [15,16]. We hypothesized that the down-regulation of the ADH and ALDH genes of the core metabolic module inhibits lactate metabolism, thereby maintaining a state of acidosis, while ensuring that lactate, with the catalysis by the up-regulation of ACSS1, generates acetyl-CoA to participate in the TCA cycle (Figure 4B). Indeed, correlation analysis indicated a significant negative correlation between the expression of ADH1A, ADH1B, ADH1C genes and lactate levels in breast cancer (Figure 4D). At the same time, the differential results of metabolite levels showed that the lactate levels in cancer samples were significantly higher than those in normal samples (*p* = 4.86 × 10^−8^; Figure 4E). The lactate accumulates as a reducing product of glucose in the tumor microenvironment during glycolysis based on the Warburg effect [17]. Lactate can serve as a metabolic substrate and signaling molecule, playing a predominant role in metabolic crosstalk between cancer cells and the tumor microenvironment (TME) [18,19]. Additionally, the core metabolic module genes were involved in the processes in which alcohol can also be catalytically converted into aldehyde and in the metabolism of fatty acids. In cancer progression, fatty acid degradation, along with lactate metabolism, represents an effective energy acquisition pathway. The differentially expressed ADH impacts the oxidation of 1-alcohol to produce aldehyde and, under the dysregulated ALDH regulation, participates in the synthesis of fatty acids, thereby exerting significant influences on cancer metabolism (Figure 4B). The combined action of ADH and ALDH, through their effects on aldehyde production and fatty acid synthesis, significantly impacts cancer metabolism, potentially altering the energy dynamics and metabolic reprogramming that are characteristic of cancer cells. Indeed, a total of 17 RNA samples were isolated from MCF7 xenograft tumors, which were co-implanted with either UCP1-CRISRPa-modulated or dCas9-VP64 (control)-treated adipose organoids in SCID mice fed with chow, high-fat diet, or 15% glucose water (GSE246231). These samples were obtained to explore the relationship between fat interference and the expression of this core metabolic module. The differential analysis revealed that ALDH2 (*p* = 0.041), ALDH9A1 (*p* = 0.045), and ADH5 (*p* = 0.041) were significantly lower expressed in high-fat diet samples than in chow diet samples (Figure S6C). To further support our hypothesis about the essentiality of these core metabolic module, we utilized publicly available CRISPR-Cas9 knockout screening data from Depmap (Dependency Map; https://depmap.org/portal/, accessed on 8 April 2026). The results revealed that, upon CRISPR-mediated knockout of ADH5 in breast cancer cell lines, the CERESs were <0 across all evaluated cell lines. Similarly, following the knockout of ALDH1B1 and ALDH7A1, over 75% of the cell lines exhibited CERESs < 0 (Figure S7A). These findings substantiate that ADH5, ALDH1B1, and ALDH7A1 play critical roles in the proliferation and survival of breast cancer cells. Additionally, ADH plays an essential role in drug metabolism involving cytochrome P450 (CYPs), such as drug metabolism-cytochrome P450 and the metabolism of xenobiotics by cytochrome P450 (Figure S5B,C). Research has elucidated the roles of ADH and CYP in ethanol pharmacokinetics through pharmacokinetic analysis [20]. Thus, the dysregulated core metabolic module genes, serving as crucial regulatory enzymes in multiple metabolic pathways, might disrupt the metabolism subpathway for glucose and fatty acids, consequently being closely associated with the energy reprogramming related to the tumor cell growth of breast cancer.

#### 2.3.3. Functional Characteristics of the Core Module Linking Risk Metabolic Subpathway

Considering the crucial role of the core metabolic module in energy and drug metabolism, we will further investigate their regulation on the progression of breast cancer. By using the GSVA algorithm to calculate the functional scores of the core metabolic module genes and dividing patients into high and low metabolic activity groups, based on these scores, significant differences in survival could also be observed between these two groups. Given that patients may receive different treatment regimens, we evaluated the prognostic value of the metabolic signature within the major treatment subgroups of the TCGA-BRCA cohort, in which 49.7% of patients were chemotherapy-naive and 22.9% received anthracycline–taxane combination regimen (AC-T) chemotherapy. For each subgroup, we stratified patients into high- and low-score groups based on the key subpathway score and performed survival analyses. As shown in Figure S7B, the metabolic signature remained prognostic in both the AC-T subgroup (n = 248, *p* =3.6 × 10^−2^) and the treatment-naive subgroup (n = 554, *p* = 3.9 × 10^−3^). Furthermore, we analyzed the differential genes between two groups, including 258 down-regulated genes and 3 up-regulated genes. The under-expressed genes in the high metabolic activity group exhibited an overlap with the key genes (ADH1A, ADH1B, ADH1C, and ADH4) of ADH in breast cancer (Figure S8A). Studies have found that ADH1B may inhibit the proliferation, invasion and migration of BRCA cells by inactivating the MAPK signaling pathway, making it a promising target for the clinical treatment of BRCA [21]. The association between ADH1C polymorphisms and the risk of breast cancer was significant in a meta-analysis of a case–control study [22]. The functional pathways enriched in the high metabolic activity group include pyruvate metabolism (*p* = 9.42 × 10^−4^), fatty acid degradation (*p* = 6.23 × 10^−4^), glycolysis/gluconeogenesis (*p* = 7.38 × 10^−4^), drug metabolism-cytochrome P450 (*p* = 1.16 × 10^−3^), and the AMPK signaling pathway (*p* < 0.05) (Figure S8B). The AMPK signaling pathway, involved in cellular energy metabolism, impacts breast cancer progression by enhancing glucose uptake and aerobic glycolysis, leading to lactate accumulation [23]. These findings are highly consistent with the regulation of metabolic pathways by the core metabolic module, further highlighting their important role in regulating metabolism in BRCA. In terms of biological processes, they are related to alcohol metabolism and immune-related functions. Therefore, in-depth exploration of this core metabolic module in the metabolic mechanisms of breast cancer holds significant clinical significance and promising potential applications.

#### 2.3.4. Subpathway Crosstalk Mediated by the Core Module Correlates with Immune Cell Infiltration in BRCA

Given the enrichment of immune-related functions in the high metabolic activity group, we further investigated the impact of the core metabolic module on immune cell infiltration. The results showed that the immune infiltration levels of multiple immune cells, including B cells naive, CD8 T cells, resting memory CD4 T cells, and monocytes, are significantly higher in the high metabolic activity group, whereas the levels of M0 macrophages, M2 macrophages, and resting mast cells are significantly lower than those in the low metabolic activity group (Figure 4F). In the GSEA enrichment results of the c7 immune-related gene set, the high metabolic activity group was significantly enriched in multiple T cell-related immune gene sets, such as GSE19198_TREATED_TCELL_UP, GSE3039_CD8_TCELL_DN and GSE36476_MEMORY_CD4_TCELL, indicating that the core metabolic module may play a crucial role in T cell immune infiltration (*p* < 0.01; Figure S8C,D). More and more evidence suggests that the immune response of tumor cells is influenced by the acidification of the tumor microenvironment (TME) caused by lactate accumulation. It is well-known that TME acidification is an immunosuppressive factor that hinders tumor immune responses [24]. Activated T cells are primarily characterized by glycolysis, which involves glucose consumption and lactate production, further contributing to lactate accumulation [25]. Therefore, we have reason to believe that the dysregulation of the core metabolic module is potentially linked to lactate accumulation and the acidification of TME, which may contribute to an immunosuppressive environment and consequently correlate with altered immune cell infiltration in breast cancer.

In conclusion, by studying the regulatory mechanisms of this core metabolic module, we would gain a deeper understanding of their roles in metabolic crosstalk and reprogramming, and further elucidate their impact on breast cancer progression and immune cell infiltration. The study of this core metabolic module in breast cancer holds great promise, providing new insights and approaches for the diagnosis and treatment of breast cancer.

#### 2.3.5. Metabolic Reprogramming of the Core Module Linking Risk Subpathways Associated with the CD8 T Cell Differentiation and Exhaustion

**Figure 5:**
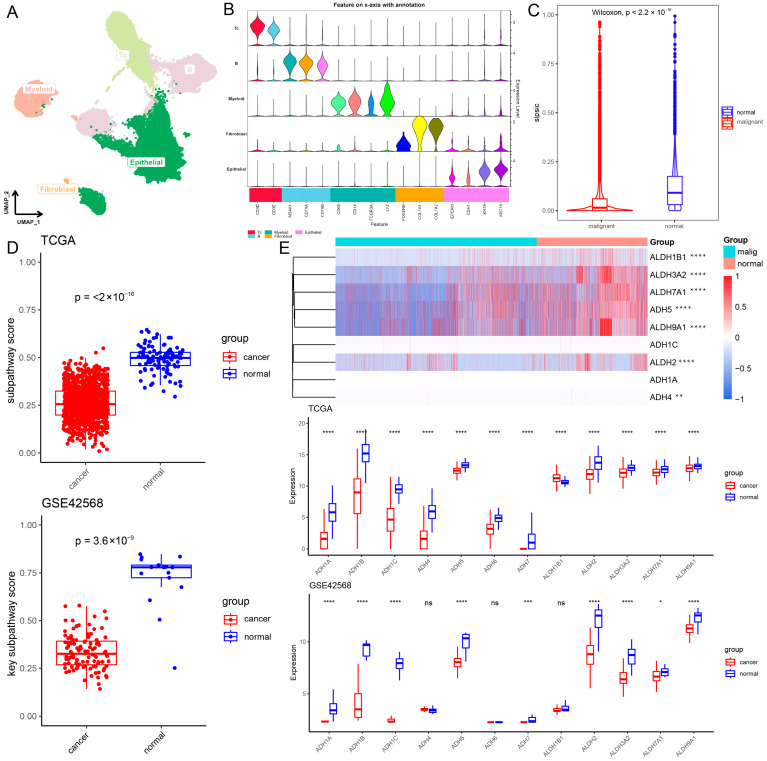
Single-cell Atlas of BRCA patients. (**A**) UMAP plots of 51,002 cells from BRCA patients, showing five major cell types. (**B**) The violin diagram shows the expression levels of selected known marker genes. (**C**) Analysis of differences in core metabolic module scores between malignant cells and normal cells. (**D**) Analysis of differences in core metabolic module scores between cancer and normal samples in TCGA-BRCA and GSE42568. (**E**) Analysis of differences in core metabolic module score genes between cancer and normal samples. ns, *p* > 0.05; *, *p* < 0.05; **, *p* < 0.01; ***, *p* < 0.001; ****, *p* < 0.0001.

To explore the mechanism of action of the core metabolic module in the breast cancer tumor microenvironment, we collected a BRCA scRNA-seq cohort (GSE195861) [26]. After quality control, 51,002 cells were contained and the cells were divided into five major cell types, including T cells (n = 12,342) identified by the expression of CD3D and CD3E, B cells (n = 11,175) which expressed MS4A1, CD79A and CD79B, fibroblasts (n = 97) marked by PDGFRB, COL1A1 and COL1A2, myeloid cells (n = 3331) identified by CD68, CD14, FCGR3A and LYZ expression, and epithelial cells (n = 24,057) which were positive for EPCAM, CDH1, KRT8 and KRT18 expression (Figure 5A,B). Next, to confirm the malignant cell subtypes, epithelial cells were further reclustered into 14 subtypes, including 7 malignant cell subtypes (0, 1, 4, 6, 7, 9, 12; n = 15,515) and 7 normal epithelial cell subtypes (2, 3, 5, 8, 10, 11, 13; n = 8542) using CNV analysis with immune cells as references [27] (Figure S9). As metabolic pathways are dysregulated in tumor cells as novel cancer markers, we used SiPSiC to establish core metabolic module scores in the breast cancer tumor microenvironment. First, analysis of the difference in these core metabolic module scores between malignant and normal cells showed that the core metabolic module scores were significantly lower in malignant cells (*p* < 2.2 × 10^−16^; Figure 5C). This suggests that this core metabolic module is less active in malignant cells, which is consistent with findings in the TCGA-BRCA and GSE42568 cohorts (TCGA-BRCA: *p* < 2.2 × 10^−16^; GSE42568: *p* < 3.6 × 10^−9^; Figure 5D). Moreover, this core metabolic module score using ssGSEA was significantly negatively correlated with tumor cells in the TCGA-BRCA, GSE42568 and five breast cancer peripheral blood cohorts (GSE67939, GSE51827, GSE111842, GSE75367 and GSE86978; R < 0, *p* < 0.05; Figure S10; Table S5). Next, investigating the genes within this core metabolic module is very important for us to further understand the role in mediating subpathway crosstalk. ALDH3A2, ALDH7A1, ALDH9A1, ADH5 and ALDH2 exhibited a significantly lower expression level in malignant cells (*p* < 2 × 10^−16^), and consistent results were also observed in cancer samples from the TCGA-BRCA and GSE42568 cohorts (*p* < 0.05; Figure 5E). Results illustrated that this core metabolic module was significantly dysregulated and its related genes had significantly lower expression levels in cancer cells and samples through the integration of single-cell and bulk data.

**Figure 6:**
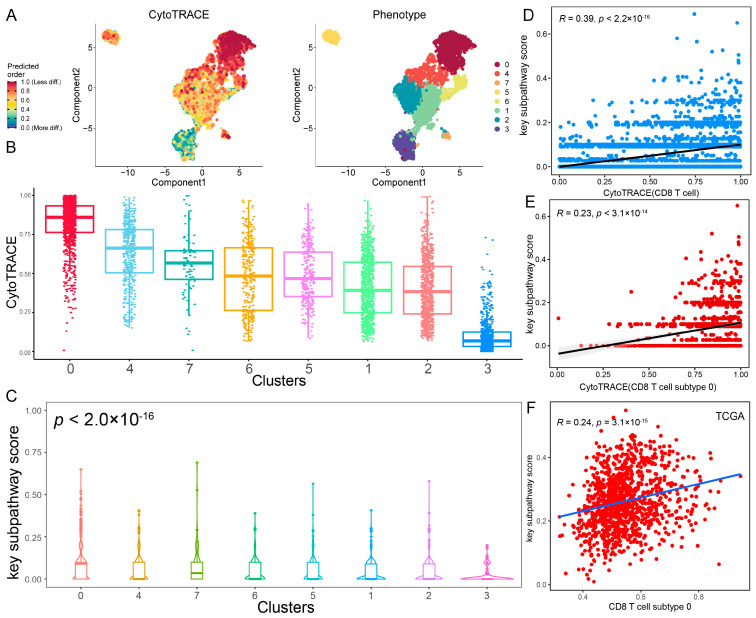
The core metabolic module is associated with the differentiation states of CD8+ T cell subtypes. (**A**) Differentiation of CD8 T cell subtypes. (**B**) Distribution of CytoTRACE scores in CD8 T cell subtypes. (**C**) Correlation between core metabolic module scores and CytoTRACE scores in CD8 T cells. (**D**) Distribution of core metabolic module scores in CD8 T cell subtypes. (**E**) Correlation between core metabolic module scores and CytoTRACE scores in CD8 T cell subtype 0. (**F**) Correlation between cancer core metabolic module scores and infiltration score of CD8 T cell subtype 0 in TCGA-BRCA cohort.

Since the core metabolic module was correlated with the immune cell infiltration of CD8 T cells, CD8 T cells were subclassified through clustering analysis into eight subtypes (Figure 6A). To reveal the origin of CD8 T cells at the single-cell level, CytoTRACE analysis was performed to decipher the differentiation state of CD8 T cell subtypes. A higher CytoTRACE score was predicted in CD8 T cell subtype 0 than in other subtypes (Figure 6A,B). The T cell subtype 0 had the highest cell stemness, representing the lowest degree of differentiation. Notably, we found that CD8 T cell subtype 0 exhibited a higher expression of exhausted T cell marker genes (PDCD1, HAVCR2, LAG3, and TOX) and a lower expression of transcription factor TCF7 (Figure S11A), which was consistent with stem-like CD8 T cells in a previous study [28]. Moreover, we observed a significant positive correlation between core metabolic module scores and CytoTRACE score in CD8 T cells (R = 0.39; *p* < 2.2 × 10^−16^; Figure 6C). Additionally, CD8 T cell subtype 0 with high cell stemness exhibited the highest core metabolic module scores (*p* < 2.0 × 10^−16^; Figure 6D and Figure S11B). Especially, we also observed a significant positive correlation between the core metabolic module scores and CytoTRACE scores in CD8 T cell subtype 0 (R = 0.23; *p* < 3.1 × 10^−14^; Figure 6E). Using ssGSEA, the core metabolic module was found to a significant positive correlation with CD8 T cell subtype 0 in the TCGA-BRCA cohort (R = 0.24; *p* < 3.1 × 10^−15^; Figure 6F). These results suggest that the core metabolic module in the tumor microenvironment may be involved in the differentiation process of CD8 T cells and affect the activation and exhaustion of CD8 T cells, which is consistent with previous studies [29,30]. Furthermore, the infiltration score of CD8 T cell subtype 0 was significantly negatively correlated with tumor cells in TCGA-BRCA (R = −0.097; *p* = 0.0016; Figure S11C) and GSE42568 (R = −0.22; *p* = 0.023) cohorts (Figure S11D). This is consistent with previous studies that stem-like CD8 T cells are critical for maintaining anti-tumor response and immunotherapy response [28,31,32]. Taken together, these results suggested that metabolic reprogramming of the core metabolic module is highly correlated with the differentiation and exhaustion of CD8 T cells. This association implies a potential impact on anti-tumor immunity, highlighting the core metabolic module as a promising therapeutic target for BRCA immunotherapy. 

## 3. Discussion

Subpathways, as the crucial components of pathways, had the potential to be biomarkers for clinical applications. In this study, we developed a novel computational approach CMSubpathway to identify cancer-related risk metabolic subpathways that were dysregulated in cancers and possessed the capabilities of cancer prognosis and classification. Firstly, differential expression analysis showed that 33.40% of the 156 metabolic subpathways were dysregulated in cancer (*p* < 0.05, Figure S12A), and 66.04% of the dysregulated subpathways were present in multiple cancer types. For instance, the subpathways hsa00620_2, hsa00071_2, and hsa00982_1 (drug metabolism-cytochrome P450), were dysregulated in more than five cancer types (Figure S12B). Further analysis revealed that a minority of dysregulated subpathways were associated with prognostic and classification efficacy, particularly in BRCA and PCPG (Figure S12C). Taking breast cancer as an example, an independent validation dataset was used to verify the robustness of the method. The discovery set and the validation set consistently identified two risk subpathways, hsa00071-2 and hsa00620-2, as breast cancer risk metabolic subpathways. Furthermore, another subpathway identification method was used to verify the robustness of the method. Consistently, one risk subpathway was identified in both the discovery set and the validation set, which had a significant intersection with the breast cancer risk subpathways. These results validated the feasibility and reliability of CMSubpathway to identify cancer-related risk metabolic subpathways.

Furthermore, we found a significant gene intersection between the two risk metabolic subpathways of breast cancer, so we regarded the intersection genes as a core metabolic module linking these subpathways. Using multiple sets of expression data and protein datasets, the genes of this core metabolic module showed consistent down-regulation at both the transcriptome and proteome levels in breast cancer patients. The core metabolic module gene ADH1A exhibited a significant correlation with breast cancer prognosis (Figure S11D; log-rank *p* = 0.032 for disease-free survival and *p* = 0.046 for overall survival). Functional analysis showed that these 12 key genes jointly influenced pyruvate metabolism, glycolysis/gluconeogenesis, and fatty acid degradation. Using paired expression and metabolite data, it was found that the metabolite lactate was significantly increased in cancer and was significantly negatively correlated with the expression of the core metabolic module genes, including ADH1A, ADH1B, and ADH1C. It is consistent with existing research showing increased lactate levels may be a marker for tumor aggressiveness, and patients with high lactate scores contribute to immune evasion and a decrease in the function of cytotoxic T cells in breast cancer [33,34]. Indeed, immune infiltration analysis showed that multiple immune cells were significantly different in the high and low metabolic activity groups. For example, the infiltration level of CD8 and CD4 T cells was significantly lower in the low metabolic activity group. Using the scRNA-seq cohort, the activity of the core metabolic module was significantly lower in malignant cells than in normal cells. Genes within this module, such as ALDH3A2, ALDH7A1, ALDH9A1, ADH5 and ALDH2 exhibited a significantly lower expression level in malignant cells and consistent results were also observed in cancer samples. The activity of the core metabolic module was significantly positively correlated with the degree of differentiation of CD8 T cells. Specifically, differential activity of this metabolic module was observed in CD8 T cell subtypes, with the stem-like CD8 T cell subtype showing the highest metabolic activity. The stem-like CD8 T cell subtype exhibited a higher expression of exhausted T cell marker genes and a lower the degree of cell differentiation. Research showed that metabolic limitations in the tumor microenvironment may exacerbate T cell exhaustion and lead to impaired anti-tumor immune responses [35]. In contrast to existing subpathway methods that rely solely on differential expression analysis or network topological structure, our CMSubpathway framework introduces a novel multi-dimensional integrative strategy that systematically identifies cancer-specific risk metabolic subpathways by integrating modeling metabolic network topology, expression dysregulation, prognostic association, and disease classification power. Beyond methodological improvements, we further revealed a novel immune–metabolic mechanism by which the core metabolic module is linked to lactate accumulation and is associated with CD8+ T cell differentiation and stem-like exhaustion. Therefore, we developed a novel method for identifying cancer risk metabolic subpathways. The identified metabolic reprogramming events were significantly associated with tumor immune cell infiltration and could act as potential biomarkers, which was conducive to the development of more effective cancer treatment strategies.

Finally, it is important to note that our findings regarding metabolic reprogramming and immune regulation are primarily based on correlative computational analyses. While these multi-omics associations are robust and consistently observed across single-cell and bulk datasets, they do not strictly establish direct causation. However, these theoretical findings can serve as a valuable theoretical foundation and be highly helpful in planning future laboratory analyses. Future in vitro and in vivo functional experiments are warranted to mechanistically validate the direct causal roles of this core module in driving metabolic reprogramming and shaping the immunosuppressive tumor microenvironment.

## 4. Materials and Methods

### 4.1. Data and Preprocessing

We downloaded the human metabolism Kyoto Encyclopedia of Genes and Genomes (KEGG) pathway KGML files from the KEGG database. A total of 84 metabolism pathway KGML files were downloaded, and the R package “KEGGgraph”(v 1.72.0) [36] was used to import and convert them into the form of interactive networks. In total, 21 different The Cancer Genome Atlas Program (TCGA) projects, each representing a specific cancer type, were analyzed. RNA-seq-based gene expression profile data were downloaded from the TCGA project. The clinical information of tumor patients, including the survival status, stage, grade, and survival time, was also downloaded from the TCGA project. The scRNA-seq data of breast cancer were downloaded from GSE195861 via the Gene Expression Omnibus database (https://www.ncbi.nlm.nih.gov/geo/, accessed on 14 March 2024) [26]. The bulk RNA-seq of breast cancer fat metabolism data were downloaded from GSE246231 via the Gene Expression Omnibus database. The bulk RNA-seq datasets of 9 breast cancer peripheral blood cohorts (including GSE111842, GSE109761, GSE111065, GSE51827, GSE55807, GSE67939, GSE75367, GSE86978, GSE41245) were collected from LncTarD 2.0 and Gene Expression Omnibus database [37]. The Seurat package (v 5.1.0) was used to load the 10X Genomics data from each sample into the R software (v 4.3.2). Using the R Harmony package (version 1.0), batch effects were eliminated based on the top 50 PCA components [38]. Based on harmony-corrected data, k-nearest neighbors (KNNs) were calculated, and a shared nearest neighbor (SNN) graph was constructed. The modular function was then modified based on the clustering algorithm to accomplish cluster recognition. The identified clusters were presented on the 2D map made with the t-distributed stochastic neighbor embedding (tSNE) or uniform manifold approximation and projection (UMAP) for the dimension reduction method [39]. For the TCGA cohort, gene expression levels were quantified as Transcripts Per Million (TPMs) and log2-transformed. For the GEO datasets, gene expression matrices were normalized using the Robust Multi-array Average (RMA) method and log2-transformed. This approach is suitable and widely applied to cross-sample comparisons, and differential expression analysis [40]. As a representative example, the TCGA-BRCA and GSE42568 cohorts were analyzed separately, and the consistently identified core metabolic module was used for subsequent analyses. Furthermore, multiple GEO datasets mentioned above were employed as independent validation sets for verification.

### 4.2. Identification of Cancer-Related Risk Metabolic Subpathways

To identify cancer-related risk metabolic subpathways (CMSubpathway), a step-by-step procedure was taken. First, the metabolic subpathways were located by means of the LTE (local tightness expansion) algorithm [41]. Second, dysregulated metabolic subpathways in cancer were identified. Third, candidate subpathways with prognostic efficacy were identified. Fourth, the classification performance of candidate subpathways was evaluated. In this study, we defined cancer-related risk metabolic subpathways that were dysregulated in cancer, correlated with prognosis, and facilitated the classification of normal and cancer samples. The schematic workflow is shown in Figure 1A. The detailed processes for the identification are as follows.

Step 1: Identification of metabolic subpathways

First, we performed a fast greedy module community recognition algorithm LTE to extract the representative subpathways from KEGG metabolism pathways. For each network, this algorithm for community detection is operated by optimizing modularity, a measure of the strength of the division of each network into communities [41]. Each node started as its community in a network, and then iteratively merged communities if two communities were adjacent to increase the overall modularity. This process continues until the modularity has no further improvement. Modularity *Q* is defined as follows:

$$Q=\frac{1}{2n}\sum_{i,j}(A_{ij}-\frac{W_{i}W_{j}}{2n})\delta (C_{i},C_{j})$$

$A_{i,j}$ is the adjacency matrix of the network (1 if there is an edge between nodes i and j, 0 otherwise). n is the total number of edges in the network. $W_{i}$ and $W_{j}$ are the degrees of nodes i and j.${\;\delta }_{i,j}$ is a function that is 1 if nodes i and j belong to the same community, and 0 otherwise. In our study, the minimum number of nodes in the module is 5, and the minimum modularity is 0.3.

Step 2: Identification of dysregulated metabolic subpathways in cancer

Second, to investigate the differences in metabolic subpathways between normal samples and cancer samples, we calculated the scores of metabolic pathways using GSEA. The R package “limma”(v 3.6.9) is used to calculate differential genes between normal and cancer samples. Differentially expressed genes are sorted in a descending rank list. For each metabolic subpathway, an enrichment score (ES) is calculated [42]. The ES reflects the degree to which the genes in the metabolic subpathway are overrepresented at the top (or bottom) of the ranked list. The formula for ES can be expressed as follows:

$$ES=\sum_{i=1}^{N}\left(\frac{r_{i}}{N}\right)$$

$r_{i}$ is the rank metric for the gene at position i of the rank list, and N is the total number of genes in which the subpathway is located. The ES is normalized to account for differences in subpathway sizes. This results in a normalized enrichment score (NES), which allows for comparison across different subpathways. To properly address multiple hypothesis testing and strictly control for false positive findings, the Benjamini–Hochberg method was applied. Furthermore, to mitigate potential biases arising from tumor heterogeneity, we implemented an iterative subsampling strategy [43]. Specifically, the process of identifying differentially expressed genes and calculating NESs was repeated 100 times. In each iteration, 70% of the total cancer and normal samples were randomly subsampled. A subpathway was ultimately defined as significantly dysregulated only if it met the criteria of abs (NES) > 1 and nominal *p*-value < 0.05 and false discovery rate (FDR) < 0.25 in at least 80 out of the 100 resampling iterations [44].

Step 3: Evaluating the prognostic efficacy of metabolic subpathways in cancer

Third, to investigate the impact of subpathways on survival, we conducted a survival analysis on metabolic subpathways. We conducted gene set variation analysis (GSVA) on cancer samples. The GSVA score for each subpathway in each sample is computed using the following formula [45]:

$$S_{ij}=\frac{1}{N}\sum_{k=1}^{N}K(M_{i}-M_{j})$$

$S_{ij}$ is the GSVA score for subpathway j in sample i. $M_{i}$ is the expression level of gene i. N is the number of genes in the subpathway. K is a kernel function (Gaussian) that defines the weighting of expression differences.

GSVA scores were used to distinguish the high-score and low-score groups, using the R function “surv_cutpoint” to find the best threshold, and then compared the survival status of the two groups. To compare survival curves between the high-score and low-score groups, the log-rank test can be used to assess whether there are significant differences between them; *p* < 0.05 was considered significant.

Step 4: Evaluating the classification performance of metabolic subpathways

Fourth, we constructed support vector machine (SVM) models to examine the classification performance of subpathways for normal and cancer samples. The SVM algorithm is widely used for cancer genomic classification and prediction [46]. We conducted five-fold cross-validation on a dataset of GSVA scores for each subpathway across various cancers in the pan-cancer TCGA dataset. We randomly divided the samples into five equal parts, selecting four for training (training set) and one for testing (test set). Each SVM model was built using the “e1071” (v 1.7.17) package in R [47] which provides an R interface to libsvm. The functions “svm()” and “predict()” were utilized to build each SVM model and to predict the sample type, respectively [48]. The outcome of the prediction was then assessed by area under the index curve (AUC) analysis. We considered subpathways with an AUC > 0.75 as candidates for classification, given that this value serves as an accepted benchmark for being considered a candidate subpathway for classification. The model has good discrimination performance [49].

### 4.3. Clustering and Cell Type Identification of scRNA-Seq

Using the “FindAllMarkers” function, we identified the marker genes for each cluster. Based on the DEGs and well-known cellular markers mentioned in CellMarker 2.0, the cell groups were annotated [50]. InferCNV (version 1.12.0) was utilized to estimate single-cell copy number variation (CNV) profiles to identify malignant cells [51]. Immune cells were used as reference cells to define a baseline of CNV estimates. Finally, compared with the reference cells, subclusters with significantly higher CNV scores were defined as malignant cells (Wilcoxon tests, *p* < 0.05, fold changes > 1.5).

### 4.4. CRISPR Knockdown

We retrieved the CRISPRGeneEffect dataset from the DepMap database (https://depmap.org/portal/, accessed on 8 April 2026). The CRISPR Essentiality Scores (CERESs) were utilized to calculate gene dependency, aiming to pinpoint genes crucial for cellular proliferation and survival. Specifically, a CERES score > 0 indicates that gene disruption enhances cell line viability, whereas a score < 0 implies that the knockout inhibits cell survival and proliferation [52].

### 4.5. Calculating the Activity of Key Metabolic Subpathways in Single-Cell RNA-Seq

The Single Pathway analysis in Single Cells (SiPSiC) (v 1.4.2) package was used to infer cancer key metabolic subpathway activity of the single-cell RNA-seq dataset [53]. We provided a list of genes contained by the cancer key metabolic subpathway, and then per-cell cancer key metabolic subpathway activity was calculated for all cells by the “getPathwayScores” function.

### 4.6. Predicting Differentiation States of T Cells from scRNA-Seq Data

CytoTRACE (version 0.3.3) is a computational framework for recreating the relative differentiation state of single-cell RNA sequencing data using the gene expression profile [54]. The differentiation state of cells may be deduced from scRNA-seq data without any previous knowledge. Initially, a KNN graph containing information on the undirected relationships between cells was built. CytoTRACE was then utilized to determine the recommended time order of cells. The KNN graph and planned time were then utilized to generate a transfer matrix, which was afterwards shown on a UMAP scatter plot.

### 4.7. Protein Level Verification

The CPTAC protein dataset was utilized to validate the results at the proteomic level, including 18 breast cancer samples and 118 normal samples. Protein expression patterns in both normal and tumor tissues were ascertained by retrieving images from the Human Protein Atlas (HPA, https://www.proteinatlas.org) database. The immunohistochemical (IHC) data of key genes in breast cancer and normal breast tissues were obtained through the HPA database.

## Data Availability

The datasets analyzed during the current study are available in the TCGA repository (https://portal.gdc.cancer.gov/, accessed on 14 March 2024), the NCBI Gene Expression Omnibus (https://www.ncbi.nlm.nih.gov/geo/, accessed on 29 March 2024) under accession numbers GSE42568, GSE195861, GSE246231, GSE111842, GSE109761, GSE111065, GSE51827, GSE55807, GSE67939, GSE75367, GSE86978, and GSE41245. The expression profile datasets E-GEOD-45581 and E-MTAB-779 were retrieved from the Expression Atlas database (https://www.ebi.ac.uk/gxa/home, accessed on 10 October 2024). Proteomic data were obtained from the CPTAC portal (https://pdc.cancer.gov/pdc/, accessed on 9 December 2024). IHC images were retrieved from the Human Protein Atlas (https://www.proteinatlas.org, accessed on 13 December 2024). CRISPR screening data were sourced from DepMap portal (https://depmap.org/portal/, accessed on 8 April 2026). Metabolic pathway information was retrieved from KEGG (https://www.kegg.jp/, accessed on 14 March 2024). Software used for analysis is public and described in detail in the Section 4. Code is available on GitHub (https://github.com/wangliTeam/CMSubpathway, accessed on 2 May 2026).

## Supplementary Materials

The following supporting information can be downloaded at: https://www.mdpi.com/article/10.3390/ijms27104246/s1.
