# Supplementary material for: Identification and Characterization of Cancer-Related Risk Metabolic Subpathways Reveal Their Functional Significance in Cancer
Source: Int J Mol Sci. 2026 May 10;27(10):4246. doi: 10.3390/ijms27104246 (PMC13207224; doi:10.3390/ijms27104246)
Supplement: Supplementary file 1 [file ijms-27-04246-s001.zip › ijms-4216128-supplementary.pdf]

Table S1 Identification of cancer-related risk metabolic sub-pathways in TCGA-BRCA data set.

Sub pathway	Survive (P)	AUC	Genes
hsa00620-2	0.049	0.8577	ALDH2, ALDH1B1, ALDH9A1, ALDH3A2, ALDH7A1, ADH1A, ADH1B, ADH1C, ADH4, ADH5, ADH6, ADH7, ACSS2, ACSS1, ACOT12, ACAT1, ACAT2, ACACA, ACACB, DLAT, ACYP1, ACYP2, AKR1A1
hsa00071-2	0.0047	0.8558	ADH1A, ADH1B, ADH1C, ADH4, ADH5, ADH6, ADH7, ALDH2, ALDH1B1, ALDH9A1, ALDH3A2, ALDH7A1, CYP2U1
hsa00330-4	0.0001	0.7882	PYCR2, PYCR1, PYCR3, PRODH, P4HA3, P4HA1, P4HA2, PRODH2, LAP3

Table S2 Identification of cancer-related risk metabolic sub-pathways in the GSE42568 data set with red-highlighted key genes.

Sub pathway	Mean (NES)	Per (P<0.05)	Survive (P)	AUC (SVM)	Genes
hsa00071-2	-1.658	1	0.017	0.762	ADH1A, ADH1B, ADH1C, ADH4, ADH5, ADH6, ADH7, ALDH2, ALDH1B1, ALDH9A1, ALDH3A2, ALDH7A1, CYP2U1
hsa00620-2	-1.755	1	0.016	0.723	ACSS2, ACSS1, ACOT12, ALDH2, ALDH1B1, ALDH9A1, ALDH3A2, ALDH7A1, ACAT1, ACAT2, ACACA, ACACB, DLAT, ACYP1, ACYP2, ADH1A, ADH1B, ADH1C, ADH4, ADH5, ADH6, ADH7, AKR1A1

Table S3 Cancer-related risk metabolic sub-pathways identified by Subpathway-CorSP in TCGA-BRCA data set with red-highlighted key genes.

Sub pathway	Survive (P)	AUC (SVM)	Genes
hsa00350-6	0.013	0.830	MAOA, MAOB, ALDH3A1, ALDH1A3, ALDH3B1, ALDH3B2, COMT, LRTOMT, ADH1A, ADH1B, ADH1C, ADH4, ADH5, ADH6, ADH7, PNMT, DBH, DDC, AOC2, AOC3
hsa00830-1	0.0011	0.858	ADH1A, ADH1B, ADH1C, ADH4, ADH5, ADH6, ADH7, BCO1, ALDH1A1, ALDH1A2, AOX1, LRAT, RDH10, RDH11, RDH5, RETSAT, RPE65, AWAT2, DGAT1, RDH12, RDH8, RDH16, DHRS9, DHRS4, DHRS4L2, DHRS4L1, DHRS3, PNPLA4, NA

Table S4 Cancer-related risk metabolic sub-pathways identified by Subpathway-CorSP in GSE42568 data set with red-highlighted key genes.

Sub pathway	Survive (P)	AUC (SVM)	Genes
hsa00830-1	0.015	0.781	ADH1A, ADH1B, ADH1C, ADH4, ADH5, ADH6, ADH7, BCO1, ALDH1A1, ALDH1A2, AOX1, LRAT, RDH10, RDH11, RDH5, RETSAT, RPE65, AWAT2, DGAT1, RDH12, RDH8, RDH16, DHRS9, DHRS4, DHRS4L2, DHRS4L1, DHRS3, PNPLA4, NA
hsa00071-2	0.0047	0.781	ACADSB, ACADS, EHHADH, HADH, ACADM, ACAT1, ACAT2, ACAA2, ACAA1, HADHB, ECHS1, HADHA, ACADL, ACOX1, ACOX3, ACADVL
hsa00280-7	0.0027	0.781	ACAT1, ACAT2, HMGCS1, HMGCS2, OXCT1, OXCT2, HMGCL, HMGCLL1, AUH, ACAA2, ACAA1, HADHB, EHHADH, HADH, ECHS1, HADHA, MCEE, PCCA, PCCB, AOX1, ALDH2, ALDH1B1, ALDH9A1, ALDH3A2, ALDH7A1, ALDH6A1, ABAT, HSD17B10, HIBADH, AACS, AGXT2
hsa00280-8	0.0084	0.762	ACAT1, ACAT2, HMGCS1, HMGCS2, HMGCL, HMGCLL1, ACAA2, ACAA1, HADHB, EHHADH, HADH, MMUT, MCEE, PCCA, PCCB, AOX1, ALDH2, ALDH1B1, ALDH9A1, ALDH3A2, ALDH7A1, ALDH6A1, ABAT, HSD17B10, HIBADH, HIBCH, ACSF3, AGXT2
hsa00280-10	0.00025	0.781	EHHADH, HADH, ACADSB, AOX1, ALDH2, ALDH1B1, ALDH9A1, ALDH3A2, ALDH7A1, ALDH6A1, ABAT, HIBADH, HIBCH, ECHS1, HADHA, ACADM, ACADS, ACAD8, AGXT2
hsa00380-5	0.031	0.800	IDO2, IDO1, CYP1A1, CYP1A2, CYP1B1, ASMT, AANAT, DDC, TPH2, TPH1, MAOA, MAOB, ALDH2, ALDH1B1, ALDH9A1, ALDH3A2, ALDH7A1, AOX1, INMT
hsa00380-6	0.0089	0.742	ASMT, AANAT, IDO2, IDO1, DDC, TPH2, TPH1, MAOA, MAOB, ALDH2, ALDH1B1, ALDH9A1, ALDH3A2, ALDH7A1, AOX1, INMT
hsa00561-2	0.022	0.804	CEL, PNLIPRP3, LIPC, PNLIP, PNLIPRP1, PNLIPRP2, PNPLA2, PNPLA3, LIPF, LIPG, MOGAT1, MOGAT3, MOGAT2, AGK, MGLL, GPAT4, GPAT2, GPAM, GPAT3, GK, GK2, AKR1B1, AKR1B10, ALDH2, ALDH1B1, ALDH9A1, ALDH3A2, ALDH7A1, AKR1A1, LPL, GLYCTK
hsa00640-4	0.0014	0.781	ALDH6A1, PCCA, PCCB, ECHDC1, ACSS3, ACSS2, ACSS1, LDHAL6A, LDHA, LDHB, LDHC, LDHAL6B, ACADM, DLD, BCKDHA, BCKDHB, DBT
hsa00650-3	0.014	0.781	OXCT1, OXCT2, AACS, HMGCS1, HMGCS2, ACAT1, ACAT2, EHHADH, HADH, ECHS1, HADHA, ACADS, ACSM1 ACSM2A, ACSM6, ACSM4, ACSM2B, ACSM5, ACSM3

Table S5 Analysis of the p value of differences in genes related to core metabolic gene module between cancer and normal samples in the datasets of the peripheral blood cohort, TCGA, and GSE42568. Red represents a P-value less than 0.05.

	TCGA-BRCA	GSE42568	GSE111842	GSE86978	GSE51827	GSE75367	GSE109761	GSE111065	GSE55807	GSE67939	GSE41245
ADH1A	<2.0e-16	1.8e-09	0.087	0.00044	0.011	0.00027	0.00034	0.00011	0.25		0.64
ADH1B	<2.0e-16	5.6e-09	0.0051	0.00028	0.00047	0.087	0.2	0.14	0.055		0.016
ADH1C	<2.0e-16	3.5e-09		0.013	0.064	0.28	0.54	0.57			0.44
ADH4	<2.0e-16	0.075	0.2	0.25	0.23	0.039	0.044	0.025	0.4		0.25
ADH5	<2.0e-16	8.4e-08	5.50e-05	1.90e-09	8.30e-05	0.00048	0.23	0.28	0.43	0.37	0.055
ADH6	<2.0e-16	0.25	0.0038	2.80e-13	3.50e-07	8.40e-10	1.50e-09	6.20e-11	0.0094		0.32
ADH7	<2.0e-16	0.00053		0.016	0.0062	0.0061	0.082	0.14	0.0048		0.67
ALDH1B1	<2.0e-16	0.06	2.70e-06	1.30e-08	2.60e-05	3.90e-08	0.91	0.43	0.036	0.011	0.082
ALDH2	<2.0e-16	4.0e-07	0.00019	2.10e-09	8.90e-07	0.0032	0.64	0.93	0.56	0.007	0.42
ALDH3A2	<2.0e-16	3.4e-07	4.20e-07	6.80e-12	7.30e-08	3.90e-09	0.015	0.044	0.9	0.41	0.039
ALDH7A1	6.9e-12	0.015	0.00068	0.68	0.032	0.093	2.10e-08	9.60e-09	0.0064	0.011	0.48
ALDH9A1	1.7e-06	1.5e-05	4.80e-07	1.60e-07	2.10e-05	3.30e-05	0.92	0.84	0.19		0.00092

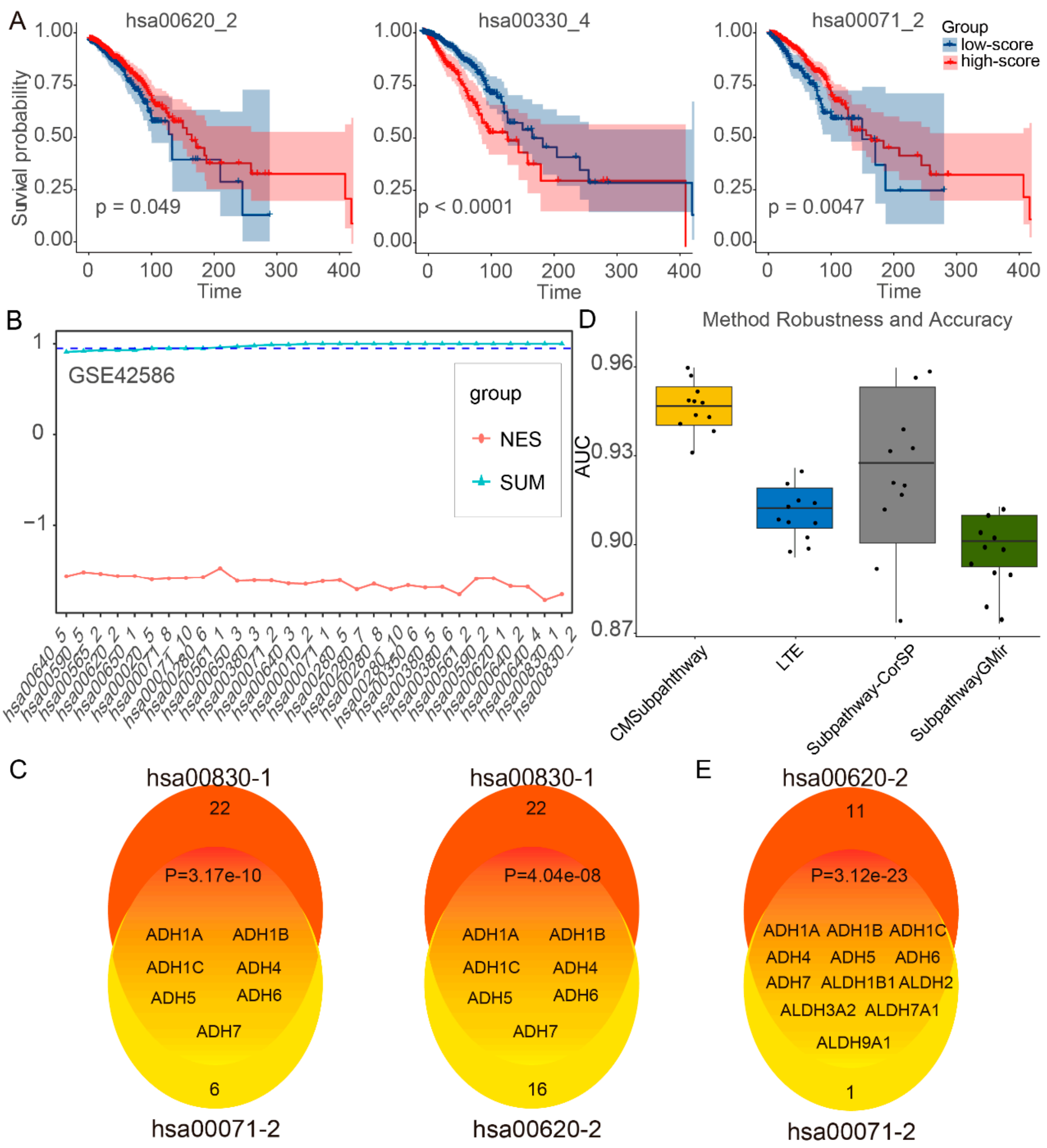

Figure S1 Independent data set validation. (A) Kaplan-Meier plots show the prognostic signal related to the risk subpathway hsa00620_2, hsa00330_4, and hsa00071_2 in BRCA. (B) The dysregulation of expression between normal and diseased samples of subpathways based on the Subpathway-CorSP algorithm in the GSE42586 data set. (C) The subpathway hsa00830_1 has a significant intersection of metabolic genes with breast cancer risk subpathways hsa00071-2 and hsa00620-2 by our method using the LTE algorithm. (D) Box plot showing the AUC scores of the four subpathway identification methods across 11 datasets, where each dot represents one independent dataset. (E) A significant intersection of metabolic genes between breast cancer risk subpathways hsa00071-2 and hsa00620-2. The 12 overlapping genes of metabolic subpathways as the core metabolic gene module.

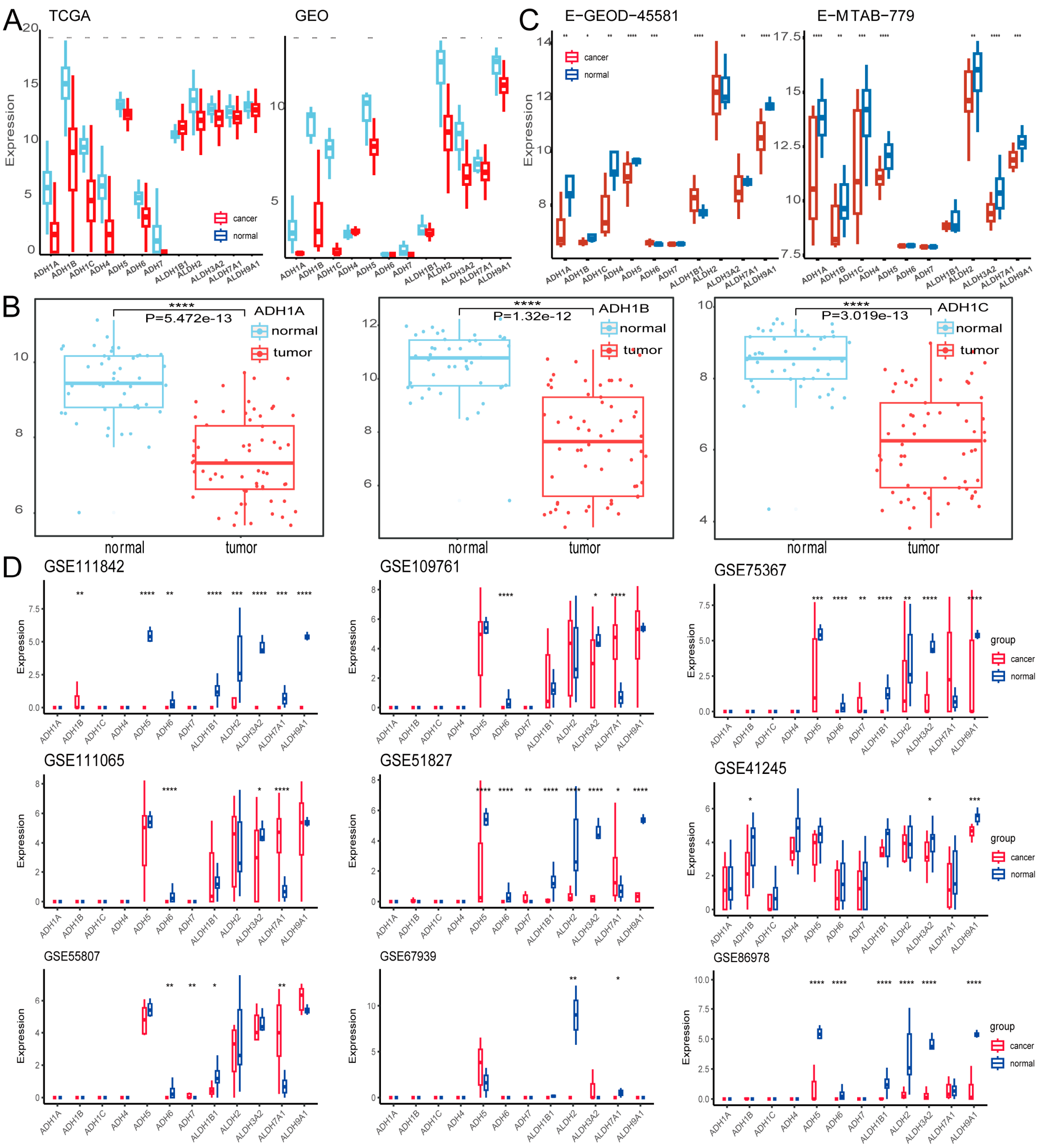

Figure S2 Dysregulation of the core metabolic gene module correlated to metabolic reprogramming (A) Analysis of differences in cancer key metabolic sub-pathway related genes between cancer and normal samples. (B) Gene expression and metabolites data of breast cancer, which encompassed 61 cancer samples and 47 normal samples from the cancer atlas of metabolic profiles (CAMP) revealed differential expression of ADH1A, ADH1B, and ADH1C in tumor and normal samples. (C) Analysis of expression levels for key subpathway genes in additional untreated breast cancer tissue cohorts. (D) Analysis of differences in core metabolic module genes between cancer and normal blood samples. Wilcoxon rank sum test were used for statistical significance. The false discovery rate (FDR) correction was applied to correct the p-value. ***: FDR< 0.001; **: FDR< 0.01; *: FDR< 0.05.

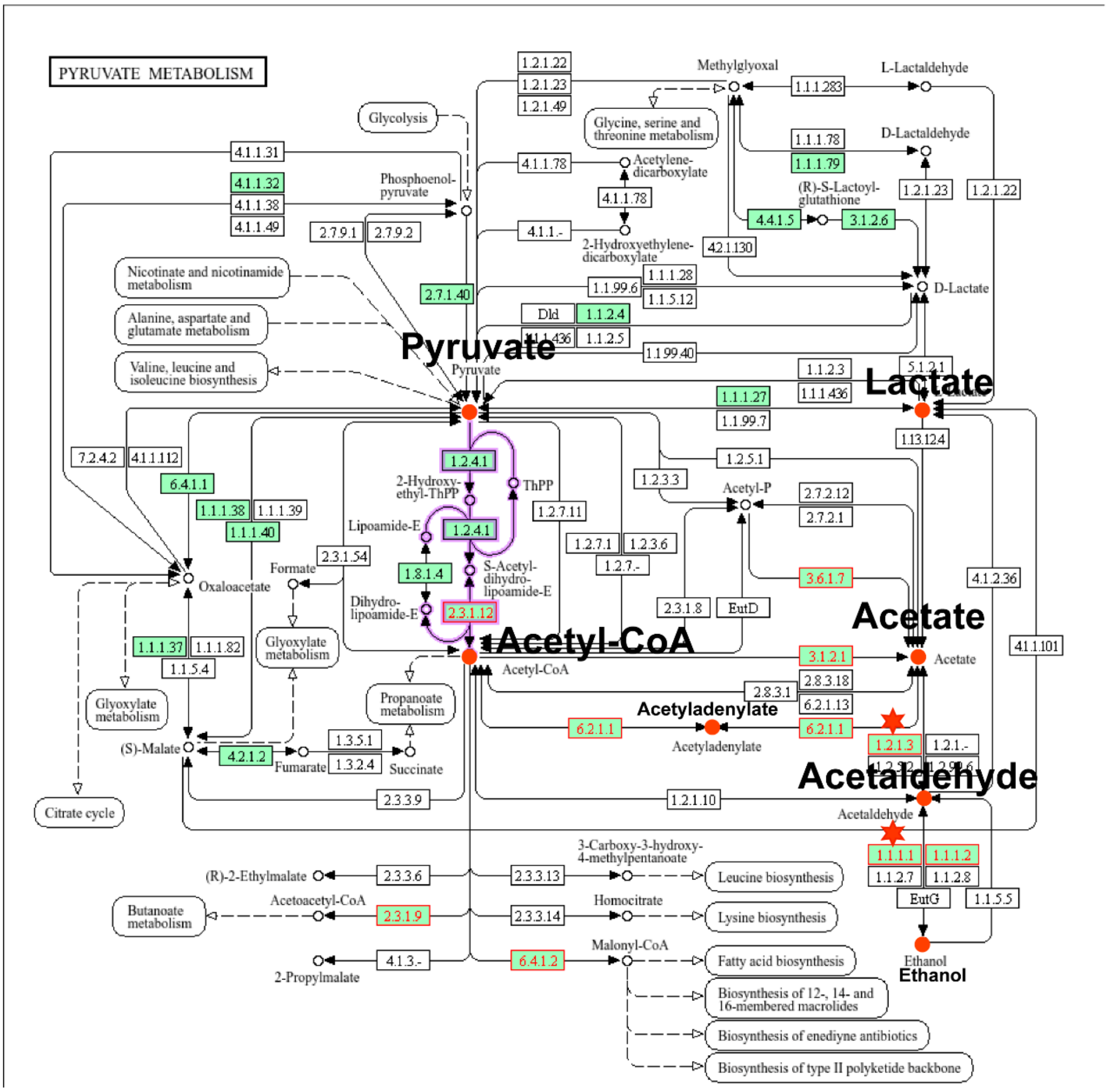

Figure S3 The regulation of key pathway gene modules in the Pyruvate Metabolism. Red circles represent key metabolites; red rectangles represent the genes invo; purple line represents the pyruvate oxidation; red hexagonal stars represent key pathway gene modules.

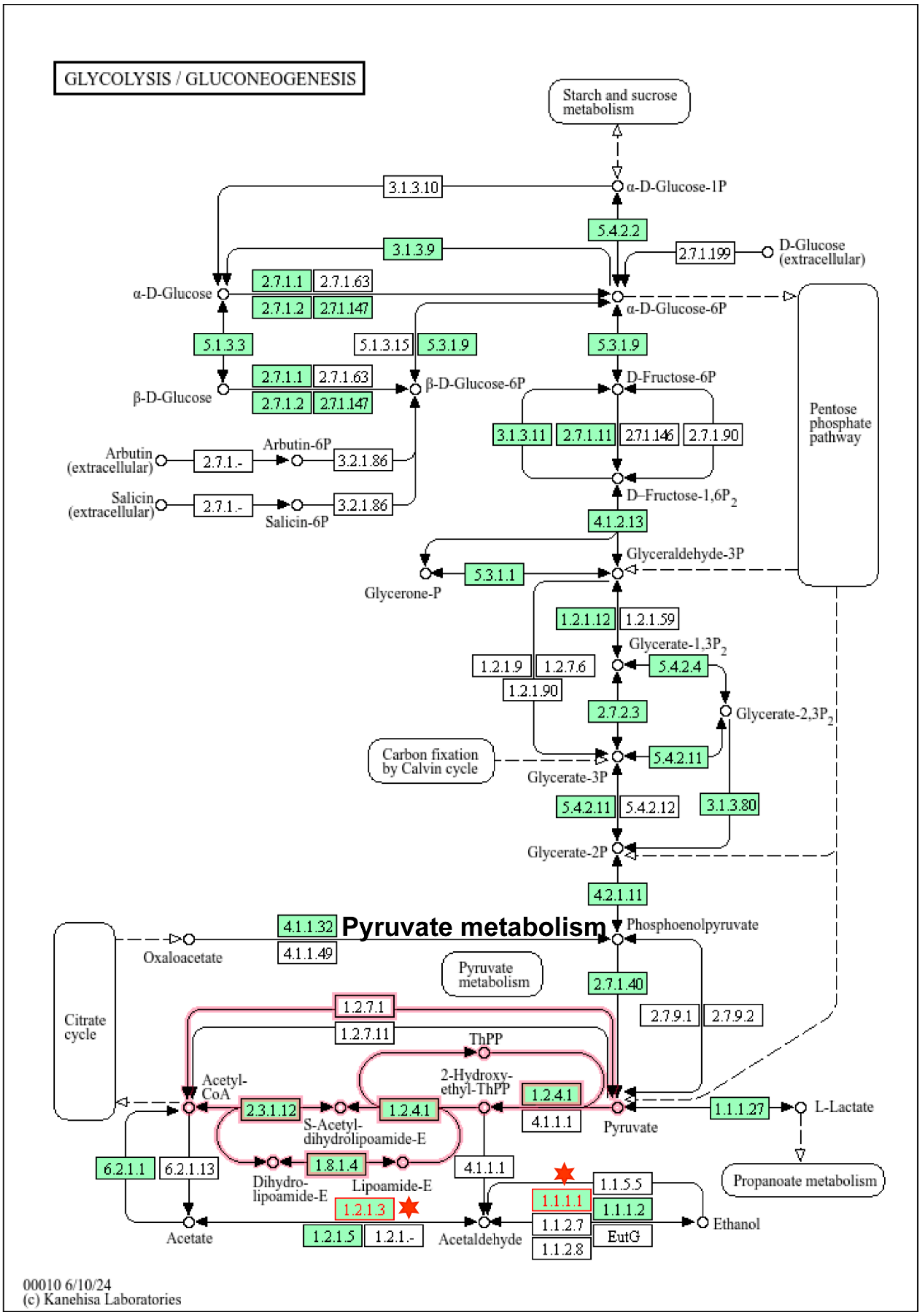

Figure S4 The regulation of key pathway gene modules in the Glycolysis/Gluconeogenesis. Red circles represent key metabolites; red rectangles represent the genes involved in two sub-pathways; red line represents the pyruvate metabolism; red hexagonal stars represent key pathway gene modules.

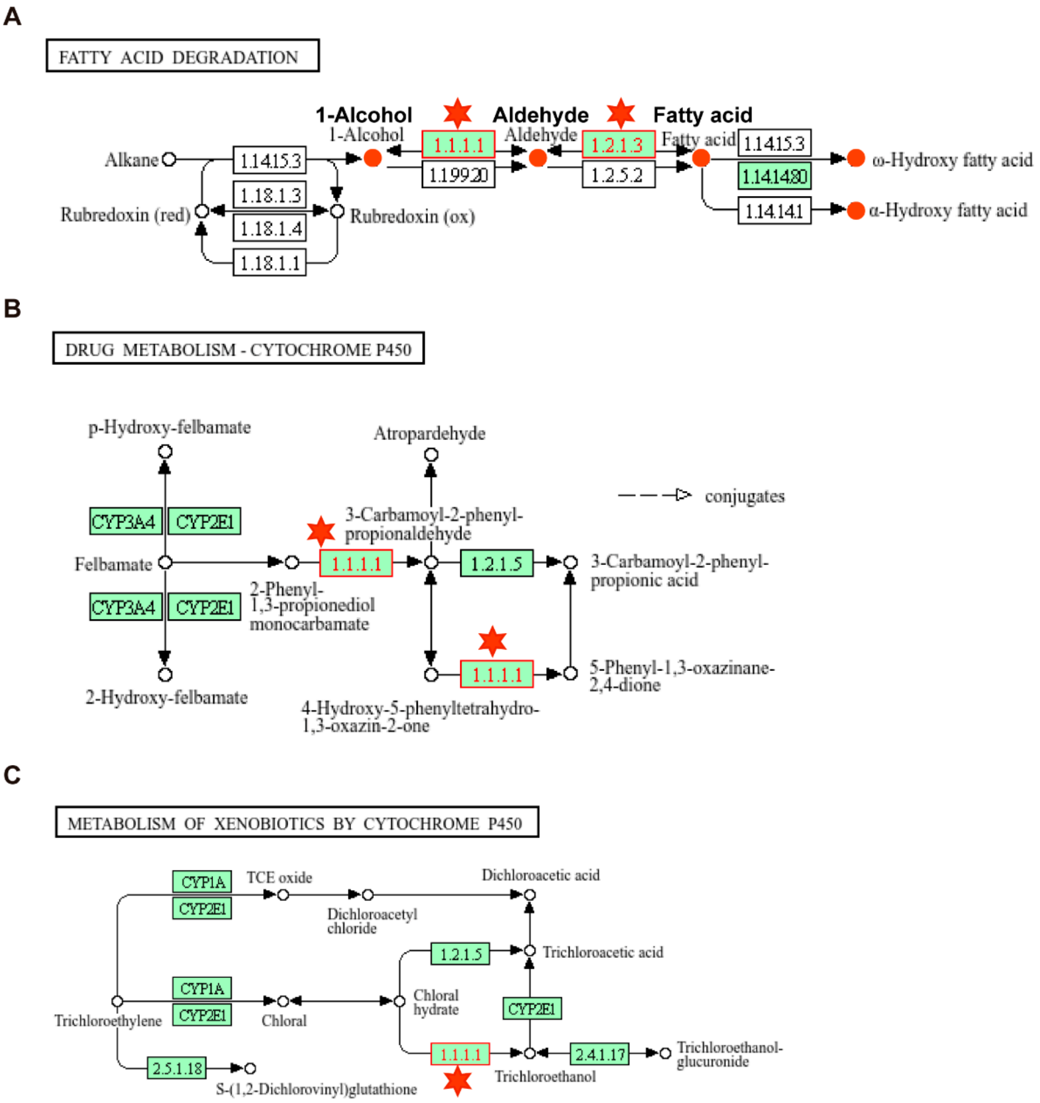

Figure S5 The regulation of key pathway gene modules in the Fatty acid degradation, Drug metabolism-cytochrome P450 and Metabolism of xenobiotics by cytochrome P450. Red circles represent key metabolites; red rectangles represent the genes involved in two sub-pathways; red hexagonal stars represent key pathway gene modules.

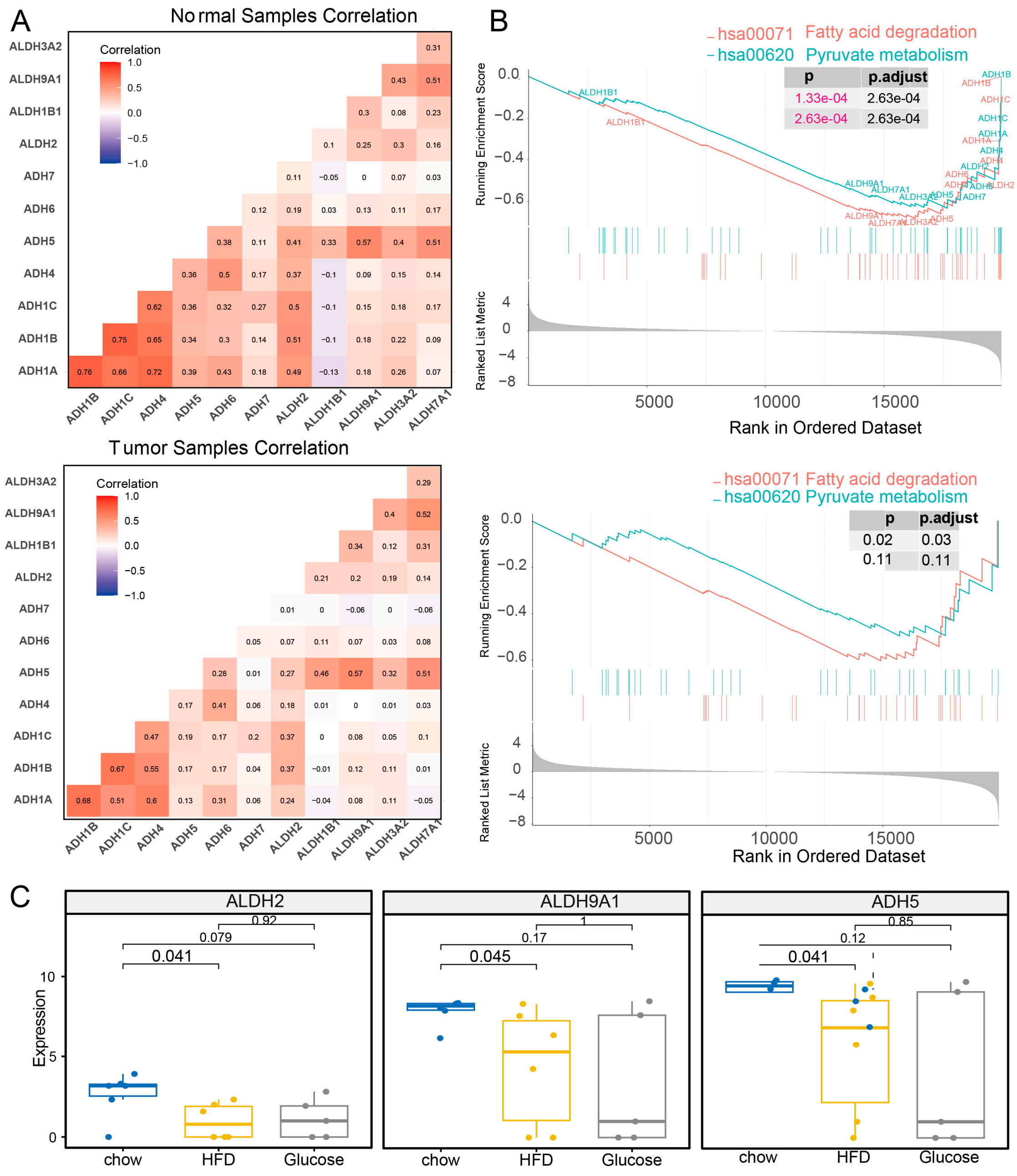

Figure S6 Metabolic crosstalk and reprogramming of the core metabolic gene module in BRCA. (A) The correlation heatmaps of key pathway gene modules in normal and tumor samples. (B) GSEA enrichment maps of differentially expressed genes in breast cancer, including or excluding 12 intersection genes from subpathways hsa00620_2 and hsa00071_2. (C) Differences in expression of ALDH2, ALDH9A1 and ADH5 between high-fat diet samples (HFD) and chow diet samples.

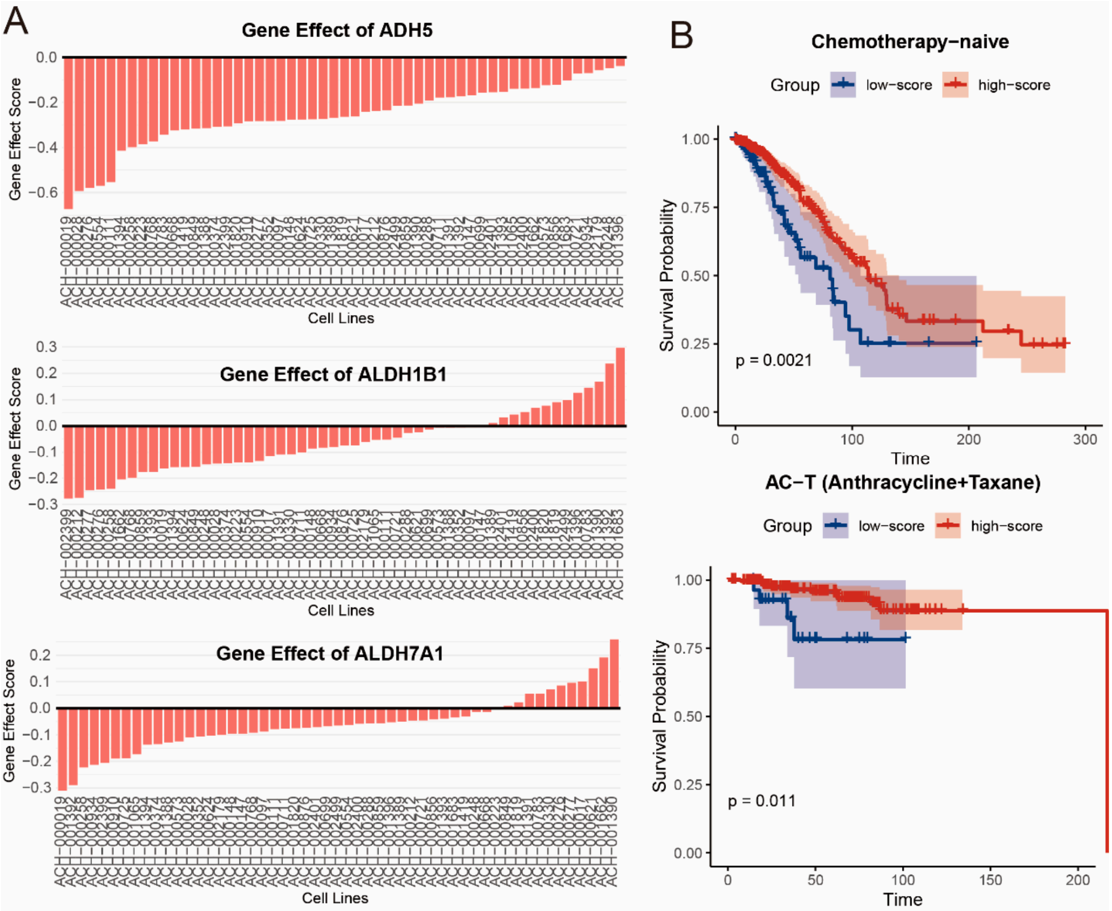

Figure S7 Dependency of key metabolic module genes in breast cancer cell lines and the prognostic significance of their score across treatment settings. (A) Gene dependency of ADH5, ALDH1B1, and ALDH7A1 in breast cancer. (B) Kaplan-Meier survival analysis of the key metabolic module score in chemotherapy-naive patients and the patient subgroup receiving the standardized AC-T regimen.

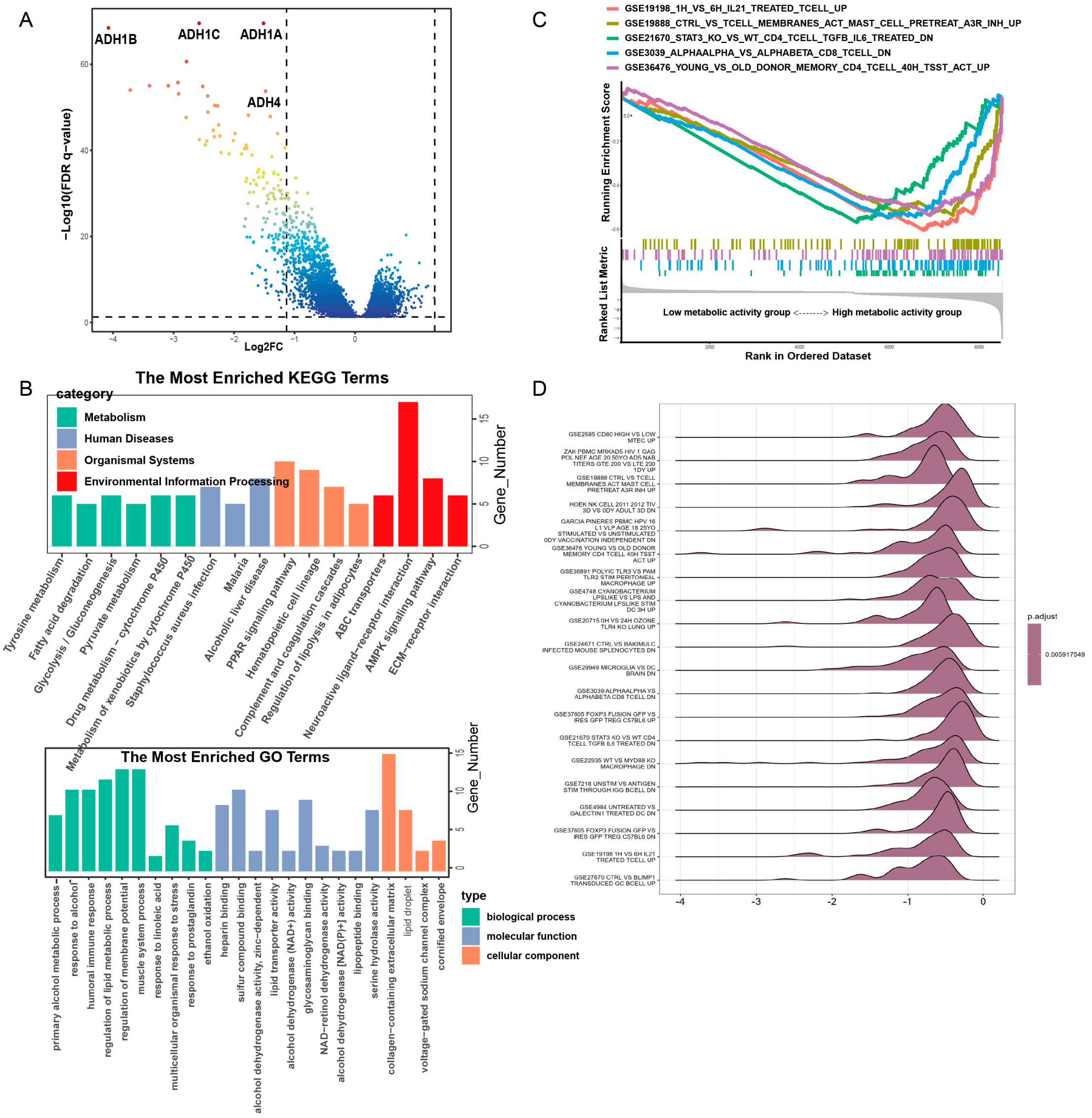

Figure S8 Core metabolic gene module impact breast cancer progression. (A) Kaplan-Meier survival analysis of the key sub-pathway score in chemotherapy-naive patients and the patient subgroup receiving the standardized AC-T regimen. (B) The volcano plot illustrates the differences in expression levels between the two sample groups. (C) Enrichment of KEGG and GO functions in the high metabolic activity group. (D) The GSEA enrichment plot shows the enrichment status of high and low metabolism activity group in T cells. (E) The GSEA enrichment results of the c7 immune-related gene set.

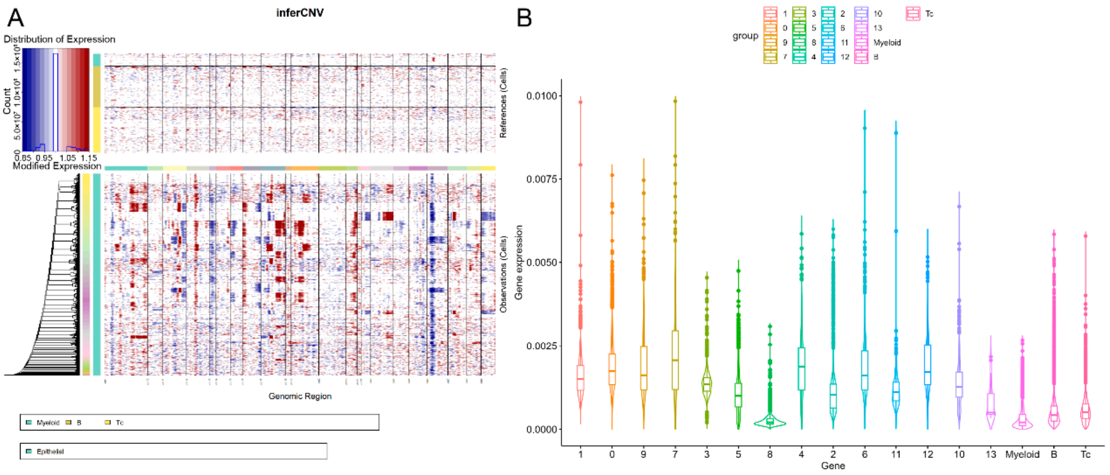

Figure S9 Deciphering the heterogeneity of cancer core metabolic gene module and functions using BRCA scRNA-seq cohort cancer. (A) The heatmap displayed large-scale CNVs of malignant cell subtypes. (B) Distribution of CNVs in malignant cell subtypes.

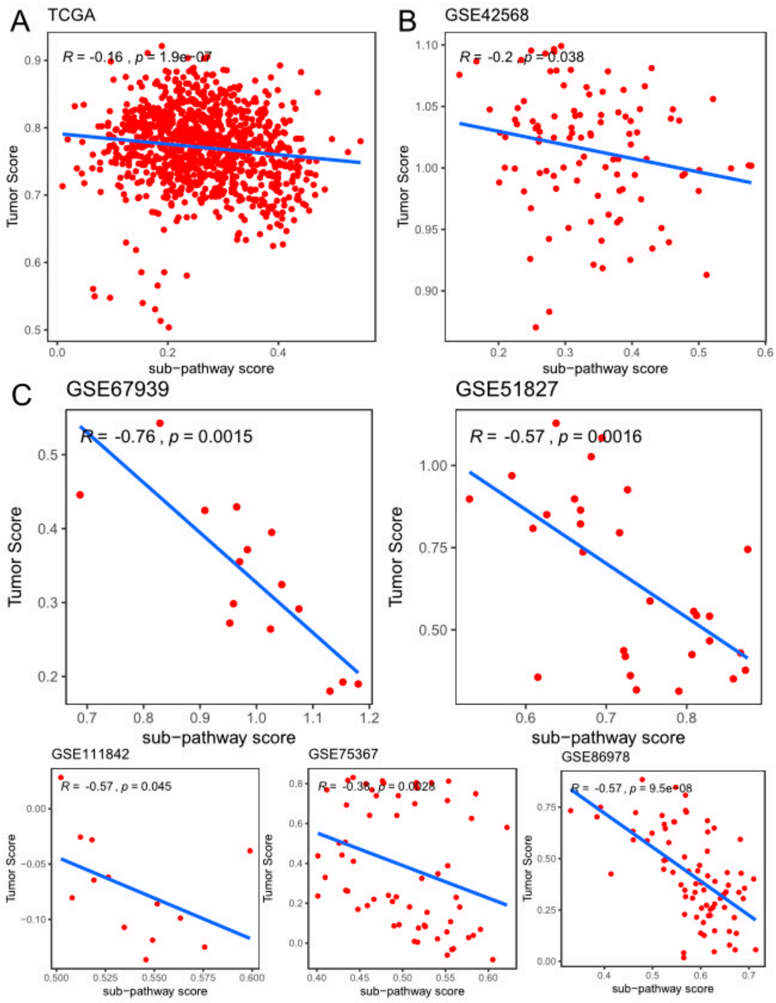

Figure S10 Correlation between cancer core metabolic gene module score and tumor cell score in TCGA-BRCA (A), GSE42568 (B) and 9 breast cancer peripheral blood cohorts (C).

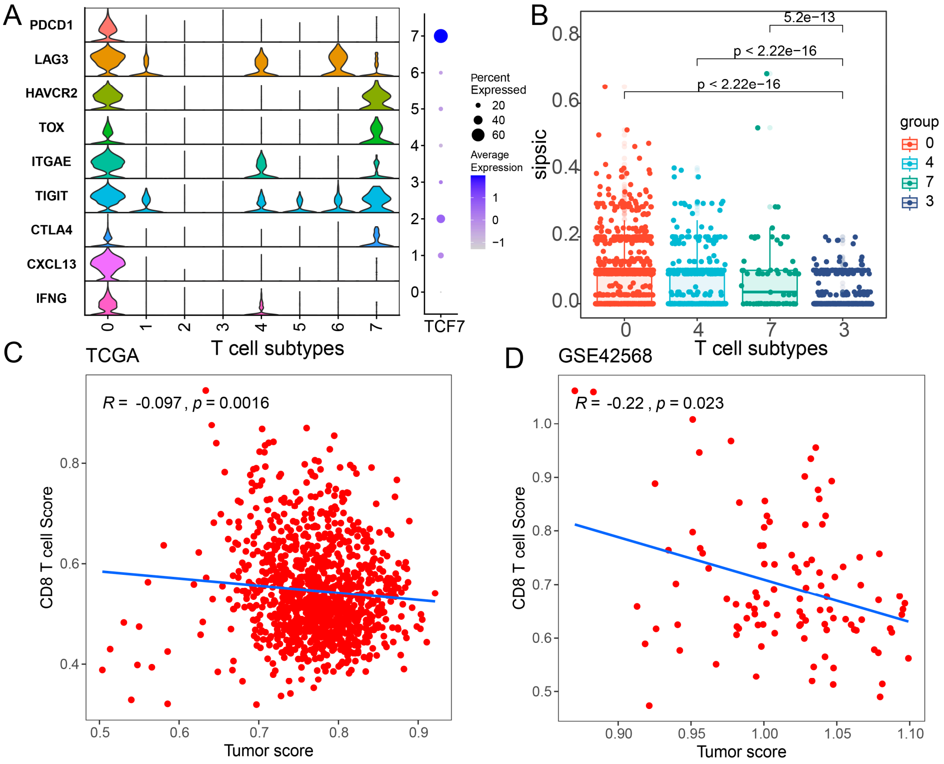

Figure S11 (A) Expression distribution of exhausted T cell markers and transcription factor TCF7 in CD8 T cell subtypes. (B) Distribution of cancer core metabolic gene module scores in CD8 T cell subtype 0, 4, 7, 3. (C) Correlation between infiltration score of CD8 T cell subtype 0 and tumor cell score in TCGA-BRCA cohort. (D) Correlation between infiltration score of CD8 T cell subtype 0 and tumor cell score in GSE42568.

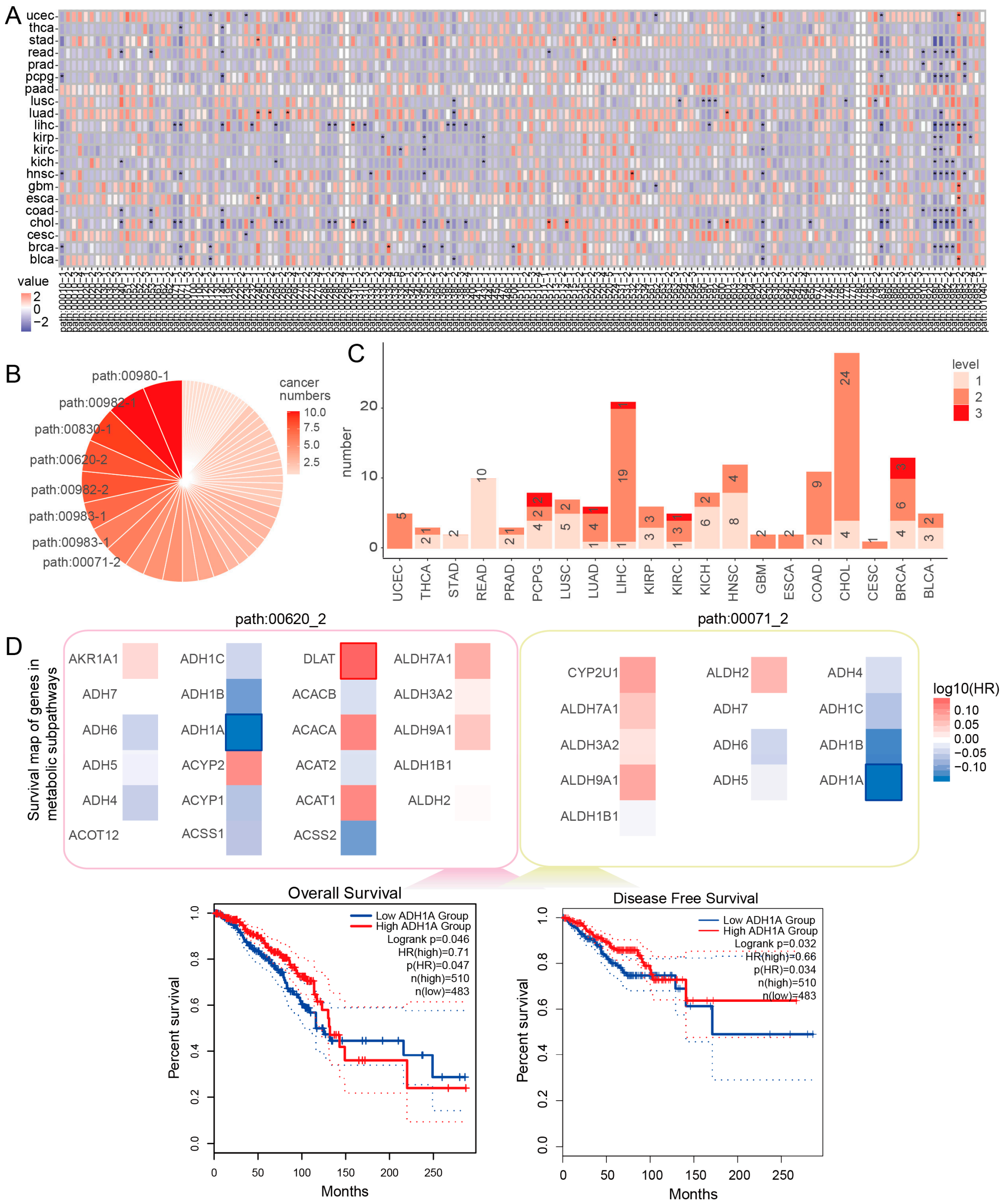

Figure S12 (A) The 156 metabolic subpathways were significantly dysregulated in 21 cancers. ***P < 0.001, ***P < 0.01, *P < 0.05. Colors represent NES scores. (B-C) Distributions of all metabolic subpathways in cancers. Includes expression, prognostic, and classification levels. (D) Prognostic value (overall survival and disease free survival) of the mRNA level of genes in metabolic subpathways in BRCA patients using GEPIA2. The upper shows the survival map of genes in metabolic subpathways; the lower shows the Kaplan – Meier plots of genes in metabolic subpathways significantly associated with BRCA prognosis (P < 0.05).
